# From zero-shot to fine-tuning: optimize large language models for error detection of ultrasound reports

**DOI:** 10.1186/s13244-026-02394-2

**Published:** 2026-09-05

**Authors:** Min Lai, Jin Zhang, Yaer Lv, Chunjie Hou, Ceng Wang, Fengzhi Li, Songcheng Xu, Meixia Du, Min Wei, Dong Xu, Litao Sun

**Affiliations:** 1 Cancer Center, Department of Ultrasound Medicine, Zhejiang Provincial People’s Hospital, Affiliated People’s Hospital, Hangzhou Medical College, Hangzhou, China; 2 Department of Ultrasound Medicine, Hangzhou Women’s Hospital (Hangzhou Maternity and Child Health Care Hospital), Hangzhou, China; 3 Department of Diagnostic Ultrasound Imaging & Interventional Therapy, Zhejiang Cancer Hospital, Hangzhou, China

**Keywords:** Ultrasonic diagnosis, Large language models, Open source, Diagnostic errors

## Abstract

**Background:**

High workload and inconsistent quality of ultrasound report writing may lead to diagnostic errors. This study aims to ascertain whether fine-tuned open-source large language models (LLMs) can achieve promising performance for automated quality control of Chinese ultrasound reports, when compared to proprietary LLMs.

**Materials and methods:**

This retrospective, multi-center study included a multi-subspecialty dataset of 1800 Chinese ultrasound reports, comprising 1500 quality-controlled reports injected artificially with six predefined error types and 300 reports with naturally occurring errors. Nine proprietary LLMs (under zero-shot and few-shot paradigms) and seven open-source LLMs (under fine-tuning) were evaluated, with performance compared against that of radiologists of varying seniority. Performance was measured by detection accuracy, Macro-F1 score, precision, recall, and mean absolute error across six categories.

**Results:**

Fine-tuned open-source LLMs, notably Qwen3-14B, achieved a detection accuracy of 0.931 and a Macro-F1 of 0.739, approaching the performance of senior radiologists. Some fine-tuned open-source LLMs maintained performance despite smaller parameter sizes and outperformed most proprietary LLMs with vastly larger parameter counts. The fine-tuned Qwen3-14B demonstrated superior recognition capability for semantic errors such as redundancy, spelling, orientation, and unit or value errors.

**Conclusion:**

This study demonstrates that task-specific fine-tuning enables open-source LLMs to rival proprietary LLMs and expert radiologists in Chinese ultrasound report error detection, offering a locally deployable and privacy-compliant alternative for AI-assisted clinical quality control workflows.

**Key Points:**

- **Question** Can task-specific fine-tuning improve error detection by open-source LLMs in Chinese ultrasound reports and provide an effective approach to automated report quality control?
- **Findings** Task-specific fine-tuning enabled open-source LLMs to achieve Macro-F1 scores up to 0.739, approaching that of experienced radiologists (0.764) and outperforming most proprietary LLMs.
- **Critical relevance statement** Task-specific fine-tuning allows open-source LLMs to become feasible assistants in ultrasound report quality control workflows, offering a locally deployable, privacy-preserving, and regulation-compliant solution for enhancing reporting consistency and reducing diagnostic errors in high-volume ultrasound examination procedures.

**Graphical Abstract:**

**Figure:**
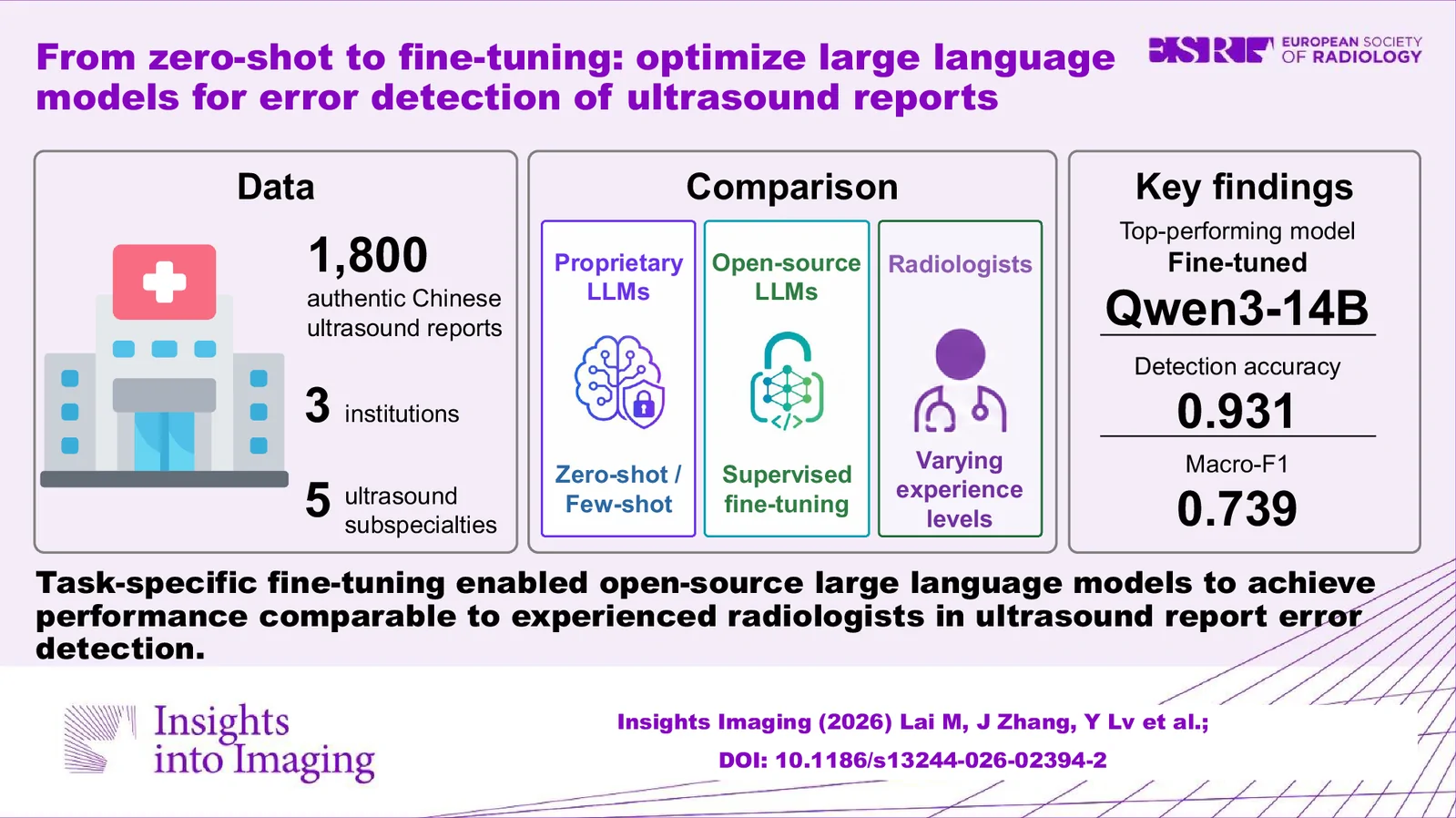

## Introduction

Ultrasonography is widely employed for the diagnosis of various organ diseases owing to its real-time visualization, cost-effectiveness, convenience, and absence of ionizing radiation. However, in most medical institutions, documentation of “ultrasound findings” and “ultrasound indications” remains predominantly reliant on templated structured reports or free-text narratives. The quality of these reports is often variable, with common issues including omissions, inconsistent terminology, and internal contradictions [1]. Concurrently, the growing volume of ultrasound examinations further increased radiologists’ workload and risk of reporting errors, which may contribute to delayed or misguided clinical decision-making and ultimately compromise patient safety [2]. Although rule-based proofreading tools have been adopted to assist ultrasound report quality control (QC) efforts, their functionality is typically confined to format validation and spell-checking, with limited capacity to capture contextual semantics or logical inconsistencies [3–5].

Recently, large language models (LLMs), such as ChatGPT, have demonstrated impressive capabilities across a range of medical natural language processing tasks, including clinical question answering [6, 7], summarization of electronic health records [8, 9], and radiology report explanation [10, 11]. Emerging studies have investigated the use of LLMs for error detection in radiology reports, reporting performance comparable to that of radiologists and suggesting a potential to significantly reduce proofreading workload [4, 12]. Despite encouraging results, the clinical deployment of proprietary (closed-source) LLMs, such as the OpenAI GPT series, remains constrained by concerns related to accessibility, transparency, data privacy, and regulatory compliance, hindering their adoption in data-sensitive or resource-limited healthcare settings [13–15]. As a result, supervised fine-tuning of open-source LLMs to improve task-specific performance and interpretability, followed by integration into clinical workflows, has emerged as a promising alternative [16–18]. Nonetheless, research on the QC of ultrasound reports remains limited, particularly in studies that encompass multiple ultrasound subspecialties and systematically evaluate the performance of various state-of-the-art LLMs. In addition, comparative assessments of open-source vs proprietary LLMs for Chinese-language ultrasound report QC tasks are yet to be established.

The objective of this study was to systematically evaluate and compare the performance of open-source and proprietary LLMs for error detection tasks in Chinese-language ultrasound reports under different inference paradigms. We hypothesized that task-specific fine-tuning of open-source LLMs would improve error detection performance compared with prompting-based proprietary LLMs in a controlled experimental setting.

## Materials and methods

### Study design and dataset

**Fig. 1:**
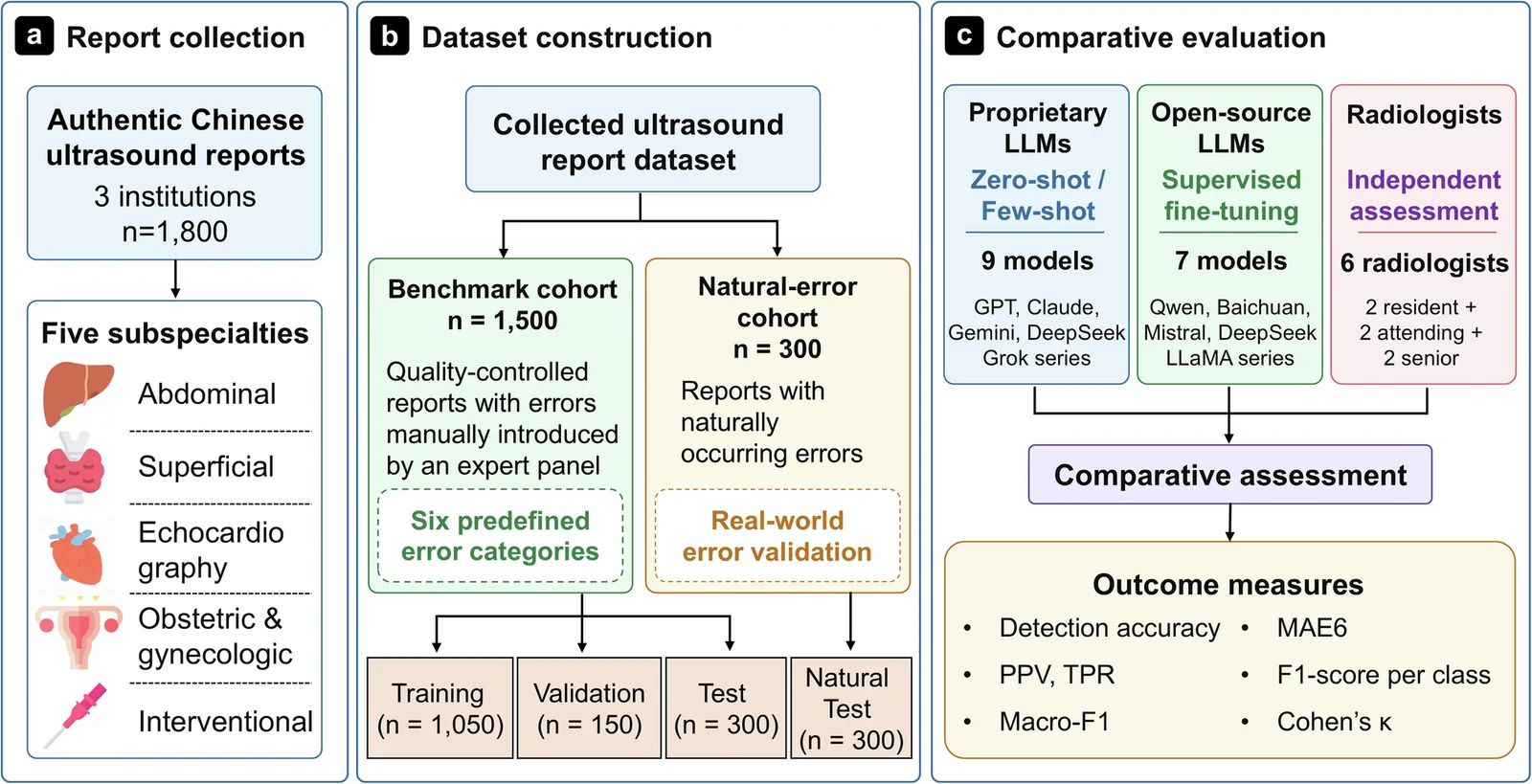
Overview of the experimental flowchart. **a** A total of 1800 authentic Chinese ultrasound reports were collected from three institutions, covering five ultrasound subspecialties: abdominal, superficial, echocardiography, obstetric and gynecologic, and interventional ultrasound. **b** The collected ultrasound report dataset was divided into a benchmark cohort of 1500 quality-controlled reports with error introduction by an expert panel and a natural-error cohort of 300 reports with naturally occurring errors for real-world validation. The benchmark cohort was further partitioned into training, validation, and primary test datasets. **c** The performance of proprietary LLMs under zero-shot and few-shot prompting, open-source LLMs after fine-tuning, and radiologists with different experience levels were compared using detection accuracy, PPV, TPR, Macro-F1, MAE6, F1-score per class, and Cohen’s κ

This study was approved by the Ethics Committees of Zhejiang Provincial People’s Hospital (IRB-2024-197), Zhejiang Cancer Hospital (IRB-2024-580(IIT)), and Hangzhou Maternity and Child Health Care Hospital (IRB-2023-184), with a waiver of patient informed consent. The overall study workflow is presented in Fig. 1. A total of 1800 authentic ultrasound examination reports from the three independent medical institutions, in either structured or semi-structured textual format, were retrospectively collected between May and December 2024. These reports encompassed five ultrasound subspecialties—abdominal, superficial, echocardiography, obstetric and gynecologic, and interventional. Among them, 1500 quality-controlled, error-free reports were used to construct the benchmark dataset, while an additional 300 reports containing naturally occurring errors were collected as an independent cohort for supplementary evaluation. All data were fully de-identified, with no patient-identifiable information retained.

Seventy percent of the 1500 quality-controlled, error-free reports were randomly selected for deliberate error introduction while maintaining balanced representation across the five subspecialties [12, 16, 19]. The error introduction was conducted by an expert panel of three radiologists, each with over ten years of clinical experience, following a standardized protocol: (1) error introduction tasks were randomly assigned to the three radiologists, with approximate balance maintained across the six predefined error types within each subspecialty; (2) each ultrasound report was manually injected with one to two errors, with error types randomly combined to replicate realistic clinical mistakes; (3) each report was annotated with a structured record detailing the error category, locations, and recommended correction; (4) each report was independently reviewed by a second radiologist for verification of error presence, linguistic consistency, and annotation accuracy, with any discrepancies resolved through discussion among all expert panel members until unanimous consensus was reached; (5) the same annotation protocol was applied to the 300 reports with naturally occurring errors; and (6) the expert panel subsequently reached consensus on all annotations, which served as the sole reference standard for model training and evaluation. To maintain objectivity, the expert panel did not participate in subsequent performance assessments.

The following six error categories were defined based on prior research to encompass the most common error types in ultrasound reports [1, 12, 16, 20]: (1) omission: the omission of clinically expected anatomical structures or lesion attribute that should be reported (e.g., missing lesion size, shape, margin, or whether the echogenicity is uniform, etc.); (2) redundancy: inclusion of irrelevant structures or unnecessary repetition; (3) unit or numerical: missing or incorrect measurement units, or implausible numeric values; (4) spelling or expression: misspellings, grammatical mistakes, or semantically incoherent phrasing; (5) logical inconsistency: lack of a coherent reasoning chain between “ultrasound findings” and “ultrasound indications”, resulting in unsupported inferences (excluding orientation errors); and (6) orientation: inconsistency in lesion location descriptions between “ultrasound findings” and “ultrasound indications” (e.g., left vs right, superior vs inferior). A constructed example illustrating all predefined error categories is provided in Appendix Fig. 1.

### Inference paradigm settings

Three representative inference paradigms were evaluated: zero-shot, few-shot [21], and supervised fine-tuning [7]. These paradigms reflect distinct capability dimensions: zero-shot understanding, in-context learning, and task-specific adaptation. In the zero-shot paradigm, the model received natural language instructions to identify and classify errors. No example ultrasound reports were provided, allowing evaluation of the model’s intrinsic generalization capability. In the few-shot paradigm, representative example reports containing known errors were embedded within the prompt to facilitate in-context learning. This setup evaluated the model’s ability to adapt to the task using limited supervision. In the fine-tuning paradigm, open-source LLMs were locally deployed and further trained via supervised fine-tuning on the structured and annotated training dataset. This approach aimed to enhance the models’ capacity to recognize predefined error types and interpret linguistic content in Chinese ultrasound reports with higher precision.

### Dataset partitioning

Given the differing dataset requirements across the three inference paradigms, the benchmark dataset of 1500 ultrasound reports was partitioned into training, validation, and test datasets in a 7:1:2 ratio for model training, example construction, and performance evaluation, respectively. In addition, the independent cohort of 300 reports with naturally occurring errors was used for supplementary evaluation. The training dataset was used exclusively to supervise fine-tuned open-source LLMs, enabling them to learn Chinese medical language patterns and error recognition. The validation dataset served two purposes: (1) supplying a sample dataset containing representative example reports for the few-shot paradigm, and (2) monitoring training performance and assessing generalization capability during fine-tuning. The test dataset was used for the unified evaluation of all LLMs and human expert groups. A double-stratified sampling strategy was applied: first by ultrasound subspecialty, then by error category within each subspecialty. This ensured balanced distribution of both subspecialties and error types across the training, validation, and test datasets.

### Procedure for proprietary LLMs

Proprietary LLMs are pretrained models whose training data, model architecture, or parameter configurations have not been publicly disclosed. These models typically possess a larger number of parameters and are trained on extensive corpora, resulting in strong generalization, robust language understanding, and generation capabilities. To comprehensively assess the current state-of-the-art LLMs for Chinese ultrasound report QC, nine high-performance proprietary LLMs were selected: the GPT series (GPT-4o, GPT-4.1, GPT-o3), Claude 3.5 Sonnet, Gemini 2.5 Pro, the DeepSeek series (DeepSeek-V3, DeepSeek-R1), and the Grok series (Grok-3, Grok-4). Detailed proprietary LLM information is provided in Appendix Table 1.

Due to the closed-source nature of proprietary models, local fine-tuning was not feasible. Therefore, two inference paradigms, zero-shot and few-shot, were adopted for evaluation. The zero-shot prompt used is detailed in Appendix Methods 1. In the few-shot paradigm, each prompt additionally contained seven annotated examples [17]: one correct and six erroneous reports. All example reports were sampled exclusively from the validation dataset, with no overlap with the test dataset. The detailed inference configuration was provided in Appendix Methods 1.

### Fine-tuning procedure for open-source LLMs

Open-source LLMs are pretrained models whose parameters, architectures, and training methods are publicly available, allowing for user-driven customization and domain-specific optimization. To improve model adaptation to the linguistic and error patterns of Chinese ultrasound reports QC tasks, seven state-of-the-art open-source LLMs with Chinese language support were selected for fine-tuning. These models varied in size (7B–14B) and architecture, including Qwen1.5-7B, Qwen3-14B, Baichuan2-7B, Baichuan2-13B, Mistral-7B, DeepSeek-7B, and LLaMA2-7B. All selected models were compatible with deployment and supervised fine-tuning on high-performance computing servers, enabling effective transfer learning from general-purpose LLMs to ultrasound reports QC applications. Detailed proprietary LLMs information is provided in Appendix Table 2. Fine-tuning was conducted using the HuggingFace framework [22] in combination with Low-Rank Adaptation (LoRA) [23], optimized for modern multi-GPU clusters to ensure both computational efficiency and model stability.

The fine-tuning training dataset consisted of 1050 reports as described previously, including 739 erroneous and 311 correct reports. Each sample was formatted as an instruction+input+output triplet. The instruction required the LLMs to determine whether the given ultrasound report contained any of the six predefined error categories. The input was a structured Chinese ultrasound report comprising ultrasound examination items, findings, and indications. The output was a standardized, six-dimensional binary vector corresponding to the six predefined error types, with each element taking a value of 1 or 0 to indicate the presence or absence of the respective error in the report. Detailed training parameters are provided in Appendix Methods 2.

### Radiologist evaluation

To evaluate the clinical applicability in ultrasound report QC, the performance of LLMs was benchmarked against that of practicing radiologists. A panel of six radiologists with varying levels of clinical experience independently performed error detection and classification on the test dataset. The panel comprised two senior radiologists (over 15 years of experience), two attending radiologists (8–9 years), and two resident radiologists (4–5 years).

### Statistical analysis

For performance comparison, each model underwent bootstrap resampling with replacement (500 iterations) based on its prediction truth pairs in the test dataset. In each bootstrap replicate, key performance metrics were calculated, including error detection accuracy, positive predictive value (PPV), true positive rate (TPR), Macro-F1 score (calculated by first measuring the F1 score for each of the six error categories separately and then averaging the six values unweighted, thereby reflecting the model’s overall ability to classify different error types while preventing more frequent categories from dominating the metric; a higher Macro-F1 indicates better and more balanced overall classification performance across error categories), and MAE_6_ (the mean absolute error (MAE) between the predicted and ground truth labels across six error categories, which reflects the average number of incorrectly predicted error categories per report and therefore provides an overall measure of how closely the predicted error profile matches the expert annotation; a lower MAE₆ indicates closer agreement with the expert annotation). Distributions of these metrics across bootstrap replicates were then compared between all possible model pairs using the two-sided Mann-Whitney U test. Performance metrics were estimated using bootstrap resampling, from which 95% confidence intervals were derived. The resulting *p*-values were visualized as heatmaps for each metric. Inter-observer agreement between radiologists and LLMs was quantified using Cohen’s κ coefficient [24]. A two-sided *p*-value < 0.05 was considered statistically significant. All statistical analyses were conducted in Python 3.12.0. Classification metrics were calculated using scikit-learn 1.3.2, and parametric, as well as nonparametric group comparisons were performed with scipy.stats 1.11.4.

## Results

### Baseline characteristics

A total of 1800 ultrasound reports were included; among them, 1500 reports formed the benchmark dataset, while 300 reports with naturally occurring errors served as the supplementary evaluation cohort. In the benchmark dataset, 1050 reports contained 1303 deliberately inserted errors. The distribution ratios of the six error categories were approximately balanced. Detailed baseline characteristics of the training, validation, test, and natural-error cohorts are provided in Appendix Table 3.

### Performance of zero-shot and few-shot proprietary LLMs

Overall, the few-shot paradigm improved detection performance for most zero-shot LLMs (ΔMacro-F1 ≈ + 0.02 to +0.07 for most models). As shown in Table 1 and Appendix Figure 2, Gemini 2.5 Pro achieved the best results, with a Macro-F1 score of 0.727 under the few-shot paradigm and 0.656 under zero-shot, showing balanced error detection performance across multiple error categories. Under the few-shot setting, Gemini 2.5 Pro achieved a detection accuracy of 0.913, PPV of 0.671, and TPR of 0.800, indicating its potential to correctly detect errors while maintaining high sensitivity. In multi-class error stability, both Gemini 2.5 Pro and Claude 3.5 had the lower MAE_6_ value (0.087 and 0.096, respectively), suggesting more consistent and reliable predictions.

**Table 1:** Performance metrics of proprietary LLMs under zero-shot and few-shot settings

	Detection accuracy		PPV		TPR		Macro-F1		MAE_6_	
Shot/model	Zero	Few	Zero	Few	Zero	Few	Zero	Few	zero	few
GPT-4o	0.839 [0.823, 0.857]	0.872 [0.856, 0.890]	0.536 [0.327, 0.609]	0.666 [0.604, 0.731]	0.267 [0.226, 0.312]	0.423 [0.366, 0.478]	0.289 [0.248, 0.335]	0.482 [0.423, 0.526]	0.161 [0.143, 0.177]	0.128 [0.110, 0.144]
GPT-4.1	0.838 [0.817, 0.860]	0.859 [0.839, 0.874]	0.657 [0.618, 0.696]	0.663 [0.602, 0.710]	0.530 [0.483, 0.588]	0.555 [0.498, 0.613]	0.515 [0.471, 0.566]	0.565 [0.515, 0.606]	0.162 [0.140, 0.183]	0.141 [0.126, 0.161]
GPT-o3	0.898 [0.887, 0.909]	0.891 [0.878, 0.905]	0.658 [0.603, 0.713]	0.616 [0.578, 0.668]	0.627 [0.580, 0.683]	0.661 [0.599, 0.711]	0.642 [0.602, 0.685]	0.635 [0.595, 0.679]	0.102 [0.091, 0.113]	0.109 [0.095, 0.122]
Claude 3.5	0.894 [0.879, 0.907]	0.904 [0.886, 0.916]	0.638 [0.589, 0.688]	0.687 [0.633, 0.746]	0.625 [0.570, 0.666]	0.648 [0.577, 0.704]	0.628 [0.585, 0.664]	0.661 [0.609, 0.706]	0.106 [0.093, 0.121]	0.096 [0.084, 0.114]
Gemini 2.5 Pro	0.903 [0.890, 0.916]	0.913 [0.900, 0.925]	0.675 [0.618, 0.727]	0.671 [0.621, 0.724]	0.648 [0.593, 0.696]	0.800 [0.768, 0.845]	0.656 [0.605, 0.698]	0.727 [0.693, 0.771]	0.097 [0.084, 0.110]	0.087 [0.075, 0.100]
DeepSeekV3	0.899 [0.886, 0.912]	0.893 [0.877, 0.909]	0.732 [0.674, 0.784]	0.716 [0.659, 0.775]	0.379 [0.326, 0.434]	0.460 [0.399, 0.512]	0.471 [0.411, 0.515]	0.547 [0.483, 0.599]	0.101 [0.088, 0.114]	0.107 [0.091, 0.123]
DeepSeekR1	0.864 [0.846, 0.879]	0.874 [0.858, 0.892]	0.615 [0.567, 0.661]	0.677 [0.621, 0.728]	0.558 [0.498, 0.610]	0.578 [0.524, 0.641]	0.564 [0.514, 0.600]	0.586 [0.529, 0.633]	0.136 [0.121, 0.154]	0.126 [0.108, 0.142]
Grok 3	0.804 [0.790, 0.822]	0.901 [0.887, 0.916]	0.184 [0.130, 0.256]	0.755 [0.717, 0.803]	0.131 [0.091, 0.176]	0.498 [0.452, 0.542]	0.151 [0.111, 0.203]	0.579 [0.530, 0.614]	0.196 [0.178, 0.210]	0.099 [0.084, 0.113]
Grok 4	0.877 [0.859, 0.889]	0.894 [0.879, 0.912]	0.647 [0.597, 0.692]	0.725 [0.682, 0.770]	0.608 [0.544, 0.660]	0.644 [0.584, 0.702]	0.607 [0.551, 0.646]	0.654 [0.603, 0.696]	0.123 [0.111, 0.141]	0.106 [0.088, 0.121]

*PPV* positive predictive value, *TPR* true positive rate, *MAE*_6_ mean absolute error across six error categories

Values in brackets represent 95% confidence intervals

### Performance of fine-tuned open-source LLMs

**Fig. 2:**
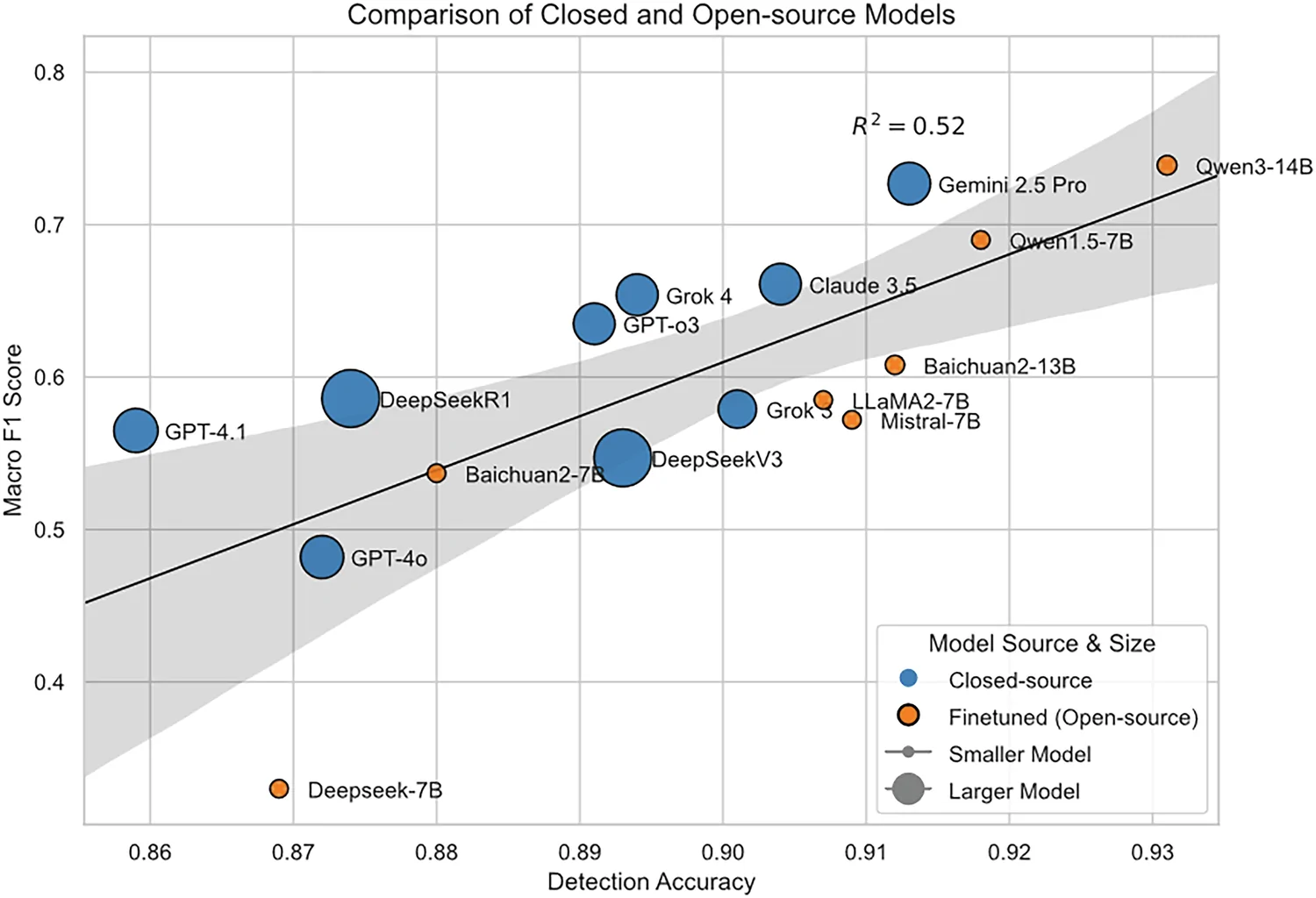
Relationship between model parameter size and error detection performance. Detection accuracy (*x*-axis) is plotted against Macro-F1 score (*y*-axis) for nine proprietary LLMs (blue circles) and seven fine-tuned open-source LLMs (orange circles). Bubble size is proportional to log₁₀(parameter count), with larger bubbles representing larger model parameter sizes. The solid black line indicates the linear regression fit, and the shaded gray area represents the 95% confidence intervals. The coefficient of determination is *R*² = 0.52

As shown in Table 2 and Appendix Fig. 3, the fine-tuned open-source LLMs generally have smaller parameter scales yet frequently outperform most proprietary few-shot LLMs (ΔMacro-F1≈up to +0.03 for most models) with parameter sizes in the hundreds of billions or even trillions. For instance, the fine-tuned Qwen3-14B achieved a Macro-F1 of 0.739, exceeding the 0.547 of the proprietary DeepSeekV3 (671 B parameters) in the few-shot setting (*p* < 0.05), reflecting marked advantages in both accuracy and error classification balance. The fine-tuned Qwen3-14B also demonstrated the greatest multi-classification stability, with the lowest MAE_6_ value (0.069) among all LLMs. Based on the overall performance comparison, we further analyzed the association between model parameter size and ultrasound report QC performance. The scatter plot (Fig. 2) demonstrated a moderate positive correlation between detection accuracy and Macro-F1 (*R*² = 0.52). However, parameter size was not a determinant of performance. The fine-tuned Qwen3-14B occupied the upper-right quadrant in both detection accuracy and Macro-F1 metrics, outperforming most proprietary LLMs with substantially larger parameter scales. In contrast, DeepSeekV3 (671 B) and GPT-4o (~200 B), despite their larger parameter scales, showed lower Macro-F1 and accuracy than several smaller LLMs. These findings suggest that, in the context of ultrasound report QC, fine-tuning smaller open-source LLMs for task-specific adaptation yields greater performance benefits than simply increasing parameter scale. The training and validation loss curves are provided in Appendix Fig. 4.

**Table 2:** Performance of open-source LLMs and radiologists in error detection of ultrasound reports

	Detection accuracy	PPV	TPR	Macro F1	MAE₆
Fine-tuned LLMs					
Qwen3-14B	0.931 [0.915,0.943]	0.783 [0.725,0.838]	0.709 [0.656,0.768]	0.739 [0.693,0.786]	0.069 [0.057,0.084]
Qwen1.5-7B	0.918 [0.902,0.930]	0.749 [0.695,0.797]	0.644 [0.598,0.696]	0.690 [0.639,0.731]	0.082 [0.070,0.098]
Baichuan2-13B	0.912 [0.902,0.924]	0.807 [0.734,0.874]	0.503 [0.459,0.576]	0.608 [0.555,0.670]	0.088 [0.076,0.098]
Baichuan2-7B	0.880 [0.864,0.897]	0.592 [0.539,0.655]	0.496 [0.449,0.562]	0.537 [0.490,0.593]	0.120 [0.103,0.136]
Mistral-7B	0.909 [0.898,0.923]	0.849 [0.782,0.915]	0.452 [0.396,0.514]	0.572 [0.515,0.628]	0.091 [0.077,0.102]
Deepseek-7B	0.869 [0.854,0.883]	0.643 [0.541,0.728]	0.230 [0.186,0.277]	0.330 [0.272,0.389]	0.131 [0.117,0.146]
LLaMA2-7B	0.907 [0.894,0.922]	0.820 [0.758,0.892	0.460 [0.404,0.526]	0.585 [0.525,0.646]	0.093 [0.078,0.106]
Radiologists					
Resident 1	0.851 [0.836,0.867]	0.716 [0.665,0.754]	0.383 [0.326,0.439]	0.439 [0.377,0.493]	0.149 [0.133,0.164]
Resident 2	0.861 [0.846,0.876]	0.577 [0.505,0.633]	0.378 [0.327,0.436]	0.448 [0.387,0.501]	0.139 [0.124,0.154]
Attending 1	0.887 [0.874,0.898]	0.621 [0.569,0.687]	0.574 [0.516,0.626]	0.589 [0.537,0.631]	0.113 [0.102,0.126]
Attending 2	0.874 [0.858,0.892]	0.677 [0.621,0.728]	0.578 [0.524,0.641]	0.586 [0.529,0.633]	0.126 [0.108,0.142]
Senior 1	0.939 [0.926,0.950]	0.874 [0.826,0.910]	0.684 [0.618,0.733]	0.764 [0.705,0.808]	0.061 [0.050,0.074]
Senior 2	0.917 [0.905,0.927]	0.820 [0.773,0.862]	0.589 [0.540,0.639]	0.675 [0.616,0.713]	0.083 [0.073,0.095]

*PPV* positive predictive value, *TPR* true positive rate, *MAE*_*6*_ mean absolute error across six error categories

Values in brackets represent 95% confidence intervals

### Performance comparison of radiologists and LLMs

**Fig. 3:**
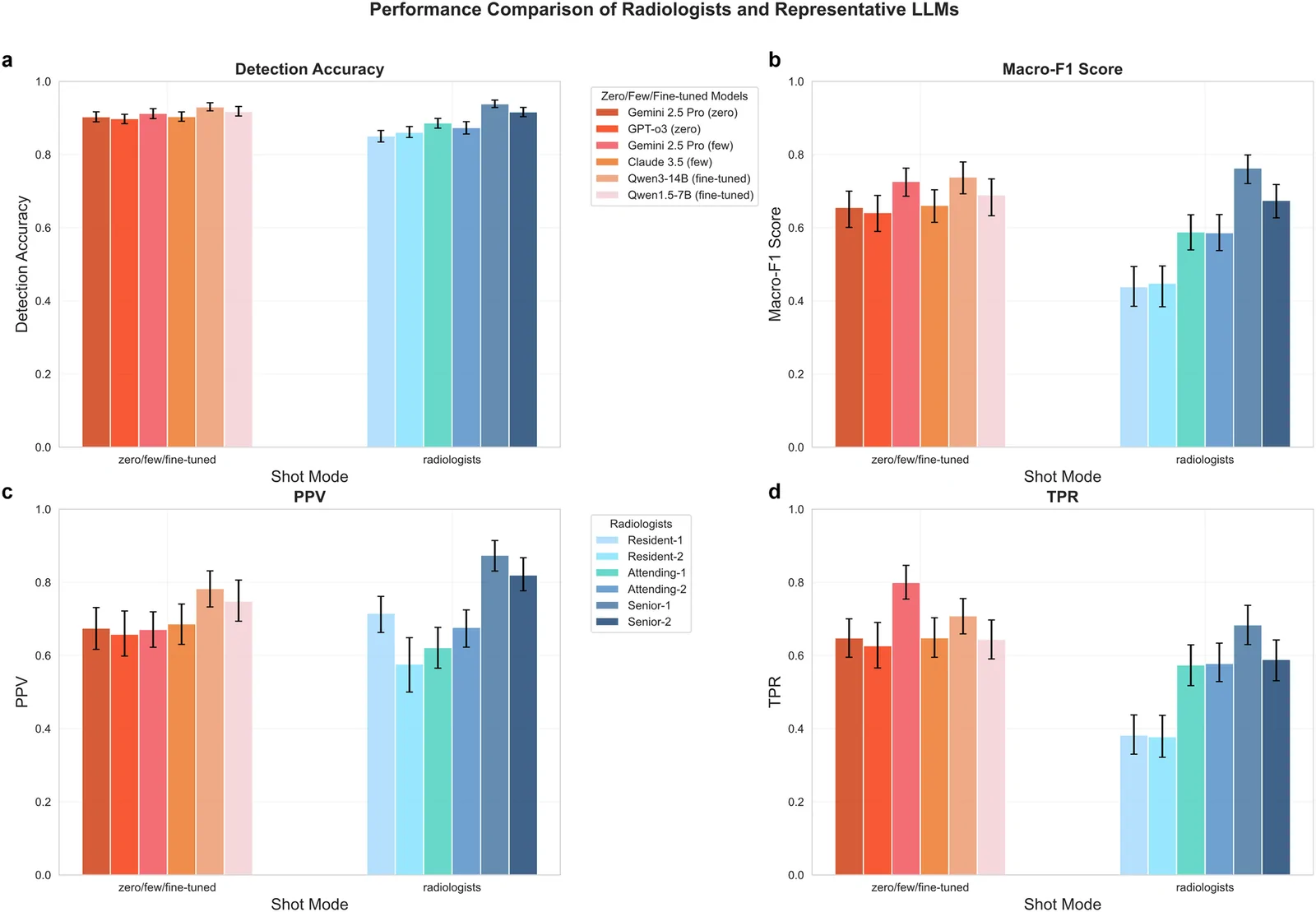
Comparison of key performance metrics between radiologists and the best-performing LLMs under each paradigm. **a** Detection accuracy; **b** Macro-F1 score; **c** PPV; and **d** TPR. Error bars indicate 95% confidence intervals

The comparative performance of the radiologists and the six top-performing LLMs under their respective paradigms is illustrated in Fig. 3, with detailed metrics provided in Table 2. The fine-tuned Qwen3-14B achieved the highest performance, showing a relatively small performance gap compared with senior radiologists under the experimental setting (ΔMacro-F1 ≈ 0.02–0.06), particularly in detection accuracy (0.931 vs 0.939, *p* < 0.05) and Macro-F1 score (0.739 vs 0.764, *p* < 0.05). In the few-shot paradigm, Claude 3.5 and Gemini 2.5 Pro ranked closely behind, achieving performance approaching that of attending radiologists in most metrics and demonstrating strong error coverage capability. These findings suggest that advanced LLMs, when applied in few-shot or fine-tuned paradigms, can achieve performance that partially overlaps with that of some mid- to high-seniority radiologists in structured error classification tasks.

The Cohen’s κ heatmaps in Appendix Fig. 5 indicate that inter-observer agreement among radiologists is higher than agreement between LLMs and radiologists. This trend is most pronounced for senior radiologists, whose κ values generally exceed 0.75. In contrast, even the best-performing fine-tuned LLMs achieved κ values of only approximately 0.6-0.7 when compared with senior radiologists. These findings indicate that, while certain LLMs are now approaching senior radiologists in aggregate metrics, there remains room for improvement in terms of consistency and stability. Finally, to provide a clinically interpretable baseline reference, we further generated a forest plot to visualize the relative stability and practical usability of each LLM across key metrics (Appendix Fig. 6). Pairwise p-values for all LLMs and radiologists are listed in Appendix Figs. 7–10.

### Performance on natural real-world errors

**Fig. 4:**
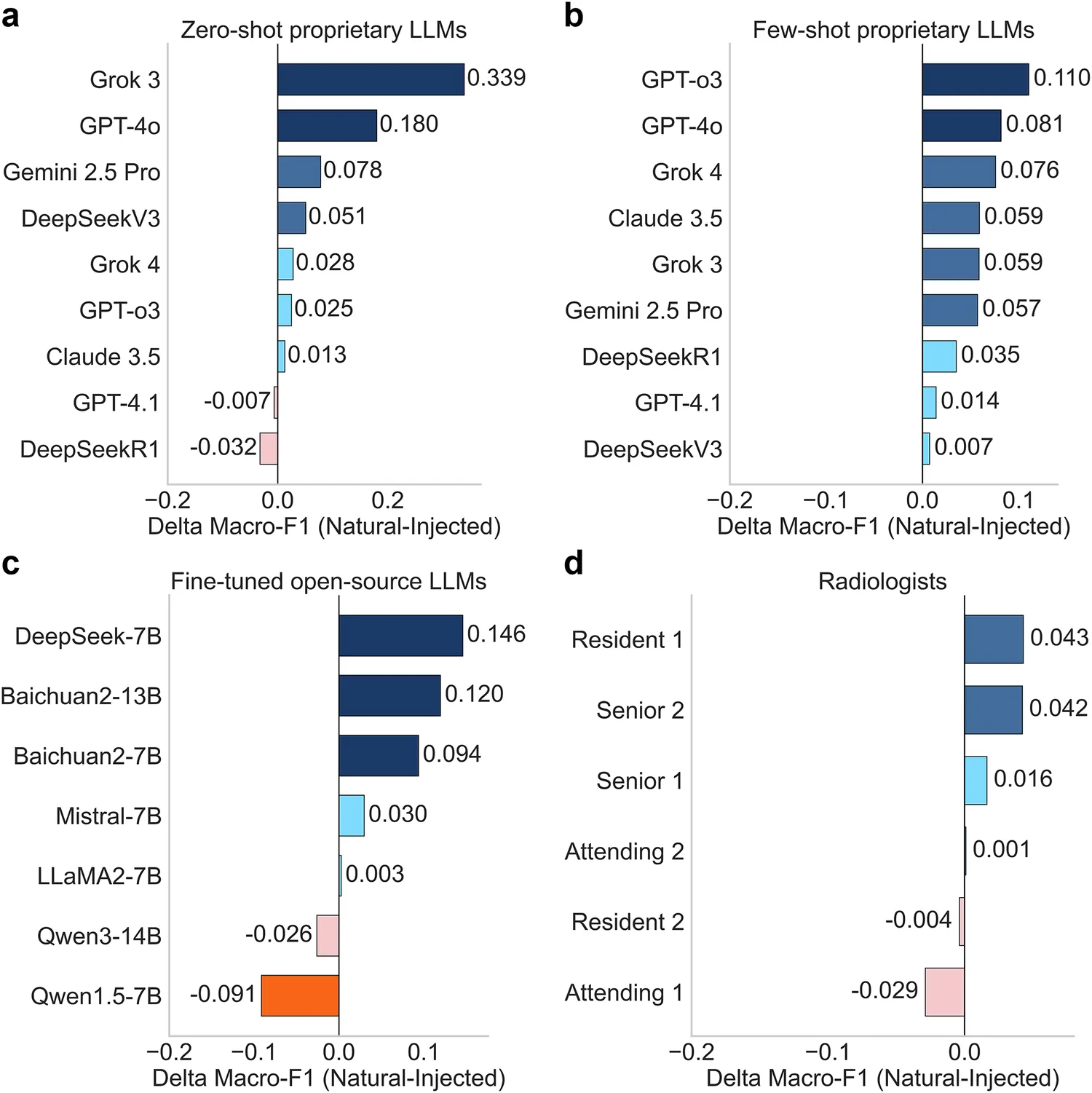
Changes in Macro-F1 performance between injected and natural real-world error cohorts. **a** Zero-shot proprietary LLMs; **b** Few-shot proprietary LLMs; **c** Fine-tuned open-source LLMs; and **d** Radiologists with different experience levels. Each bar represents the difference in Macro-F1 score between the natural-error cohort and the injected-error benchmark (delta Macro-F1 = natural-injected). Positive values indicate improved performance on natural errors, whereas negative values indicate performance degradation. Values adjacent to each bar denote the magnitude of change

Performance in the natural-error cohort showed heterogeneous changes compared with the artificial benchmark cohort (Fig. 4 and Appendix Table 4). Among proprietary LLMs, Gemini 2.5 Pro remained the best-performing model in both zero-shot and few-shot settings, with Macro-F1 scores of 0.734 and 0.784, respectively, followed by GPT-o3 with 0.667 and 0.745. Several fine-tuned LLMs demonstrated improved performance on natural errors, including Baichuan2-13B, which increased from 0.608 to 0.728, and DeepSeek-7B, which increased from 0.330 to 0.476. In contrast, Qwen3-14B showed a modest decrease from 0.739 to 0.713. Fine-tuned LLMs and few-shot proprietary LLMs continued to outperform zero-shot LLMs, while senior radiologists achieved the highest performance, showing relatively stable results across datasets.

### Performance across different error categories

**Fig. 5:**
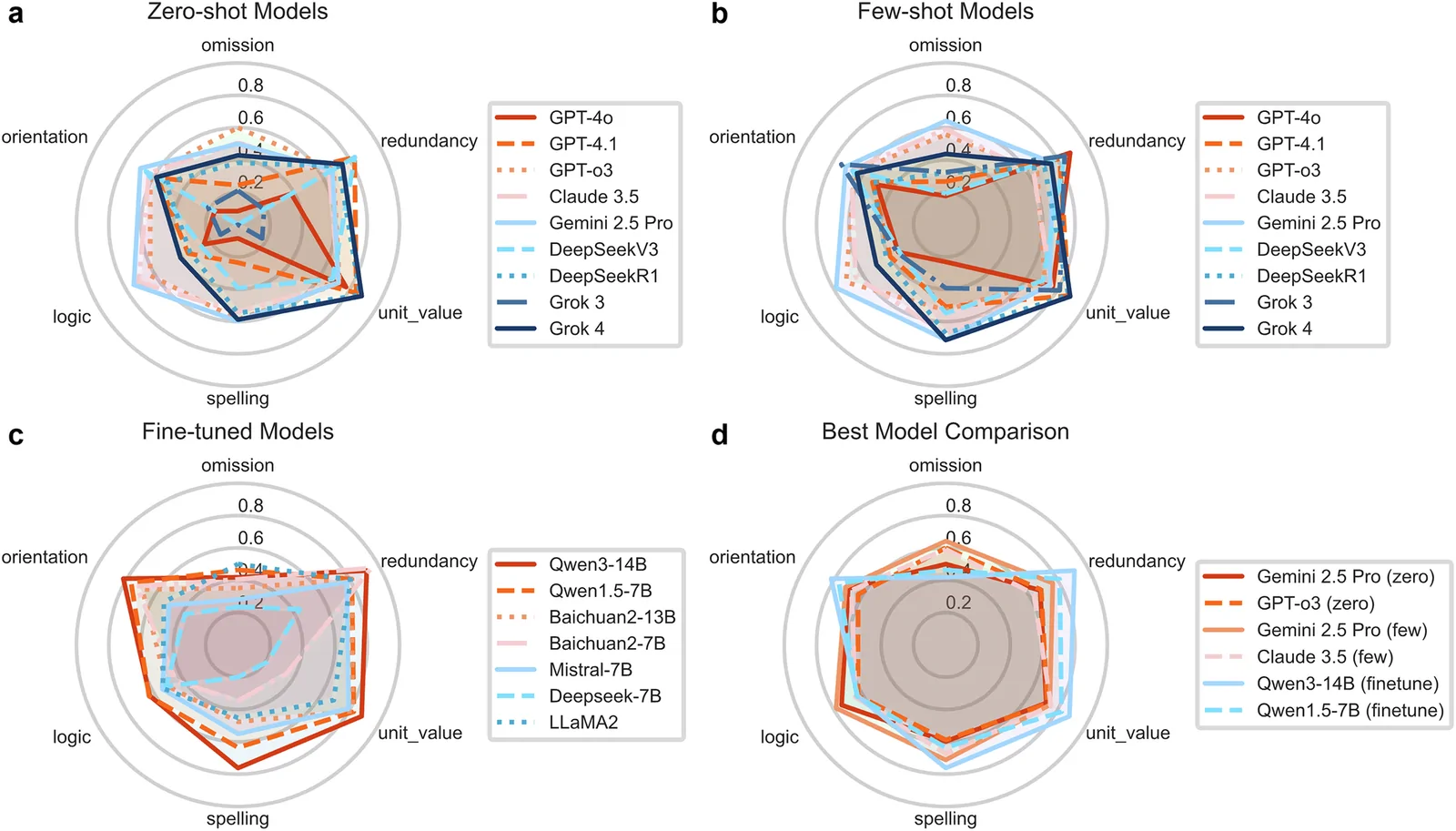
Radar plots illustrating model performance in identifying six error categories. **a** Zero-shot paradigm; **b** Few-shot paradigm; **c** Fine-tuned paradigm; and **d** Cross-comparison of the top-two performing models within each paradigm. The contour area of the radar plots represents the model’s ability to detect the six error categories: the larger the enclosed area and the closer the data points are to the outer circumference, the stronger the detection capability for that error type. Line styles and colors in the legend correspond to individual models, highlighting their respective performance profiles and detection preferences

To further investigate differences in the ability of LLMs to identify specific error categories under the three inference paradigms, radar charts were generated to visualize the distribution of performance across error types. The F1-score was used as the performance metric. As shown in Fig. 5a–c, the few-shot paradigms generally improved multi-dimensional performance compared with zero-shot, substantially enhancing the ability to capture fine-grained error categories. Several fine-tuned LLMs, especially Qwen3-14B, demonstrated superior recognition capability for semantic errors such as redundancy, spelling, orientation, and unit or value errors, whereas some few-shot LLMs exhibited greater sensitivity to logic errors. Using the Macro-F1 score as the benchmark metric, the two highest-scoring LLMs from each of the three inference paradigms were selected for comprehensive comparison. The detailed F1 scores for each error category across the six highest-scoring LLMs are provided in Table 3, with corresponding radar plots shown in Fig. 5d. The fine-tuned Qwen3-14B achieved the highest overall performance, with F1-scores of 0.418, 0.920, 0.884, 0.762, 0.630, and 0.819 for omission, redundancy, unit/value, spelling, logic, and orientation errors, respectively. Box plots comparing the F1-scores for the six error types across LLMs are provided in Appendix Figs. 11–14. In addition, Venn diagrams were constructed to illustrate the similarities and differences in error predictions among three top-performing LLMs in each paradigm (Appendix Fig. 15).

**Table 3:** F1-scores for the classification of different error types achieved by the best-performing models under each inference paradigm.

		Omission error	Redundancy error	Unit/value error	Spelling error	Logic error	Orientation error	Macro-F1
Zero-shot	GPT-o3	0.600 [0.461, 0.722]	0.682 [0.576, 0.777]	0.725 [0.605, 0.811]	0.588 [0.482, 0.707]	0.628 [0.489, 0.734]	0.627 [0.516, 0.734]	0.642 [0.602, 0.685]
Zero-shot	Gemini 2.5 Pro	0.500 [0.341, 0.635]	0.675 [0.539, 0.778]	0.718 [0.594, 0.807]	0.597 [0.442, 0.693]	0.745 [0.637, 0.841]	0.698 [0.604, 0.795]	0.656 [0.605, 0.698]
Few-shot	Claude 3.5	0.592 [0.426, 0.708]	0.629 [0.516, 0.731]	0.753 [0.624, 0.851]	0.675 [0.536, 0.768]	0.646 [0.521, 0.741]	0.674 [0.568, 0.765]	0.661 [0.609, 0.706]
Few-shot	Gemini 2.5 Pro	0.642 [0.518, 0.766]	0.764 [0.692, 0.846]	0.743 [0.631, 0.828]	0.712 [0.612, 0.810]	0.784 [0.696, 0.861]	0.716 [0.623, 0.820]	0.727 [0.693, 0.771]
Fine-tuned	Qwen3-14B	0.418 [0.266, 0.551]	0.920 [0.864, 0.967]	0.884 [0.814, 0.944]	0.762 [0.624, 0.852]	0.630 [0.497, 0.722]	0.819 [0.738, 0.909]	0.739 [0.693, 0.786]
Fine-tuned	Qwen1.5-7B	0.464 [0.336, 0.593]	0.814 [0.730, 0.884]	0.828 [0.737, 0.886]	0.635 [0.474, 0.741]	0.635 [0.503, 0.742]	0.763 [0.682, 0.870]	0.690 [0.639, 0.731]

Values in brackets represent 95% confidence intervals

## Discussion

This study systematically evaluated the performance of state-of-the-art proprietary and open-source LLMs in the Chinese ultrasound reports QC task, encompassing three representative inference paradigms: zero-shot, few-shot, and fine-tuning. The results showed that proprietary LLMs, particularly Gemini 2.5 Pro and Claude 3.5, demonstrated stable performance in overall detection accuracy and Macro-F1 score. In the zero-shot setting, some proprietary LLMs achieved performance approaching that of resident radiologists. The few-shot paradigm further improved performance, notably enhancing the ability to detect omission and spelling errors. After localized supervised fine-tuning, several open-source LLMs (e.g., Qwen3-14B and Qwen1.5-7B), despite having substantially fewer parameters than proprietary LLMs, achieved performance across multiple metrics approaching that of senior radiologists. These findings suggest that, compared with relying solely on in-context learning with general-purpose proprietary LLMs, task-specific fine-tuning of open-source LLMs can provide greater and more controllable gains in error detection for ultrasound reports [25, 26].

In previous research on LLMs in the field of radiology, researchers reported that GPT-4 achieved an overall error detection rate of 82.7% in English radiology reports, comparable to radiologists and potentially reducing workload [12]. This indicates that general-purpose proprietary LLMs can already deliver high efficiency and accuracy in error screening of radiology reports, in line with the findings of this study. Similar results have been reported by other groups [1, 27], although these studies mainly focused on QC of radiology reports in English contexts (e.g., X-ray, CT, MRI) and typically evaluated proprietary LLMs in few-shot settings. In the more linguistically and semantically demanding context of Chinese ultrasound reporting, multi-center research [20] has shown that in few-shot prompting, Claude 3.5 outperformed senior radiologists overall, while GPT-4o excelled in detecting spelling errors, with substantial variability in detection rates across error types. These findings highlight that both task characteristics and language context can markedly influence LLMs' performance. However, no previous studies have directly compared proprietary and open-source LLMs for ultrasound report QC, particularly the most recently updated LLMs.

When the weights of proprietary LLMs cannot be modified, the few-shot paradigm remains the most cost-effective and practical approach for task adaptation, substantially enhancing error detection performance. Nevertheless, its effectiveness heavily depends on the relevance and specificity of the examples included in the limited-length prompt, which constrains its iterative optimization in real-world clinical workflows. Moreover, proprietary LLMs typically require remote API calls, which may introduce latency, instability, and potential data security concerns [11]. Recent studies [16, 28] indicate that, although proprietary LLMs retain a performance advantage, privacy-preserving open-source LLMs can match the GPT series across diverse medical text tasks, while enabling on-premise deployment and compliance governance. Furthermore, fine-tuned LLaMA-3-70B-Instruct has been shown to improve radiology report error detection over general-purpose proprietary LLMs, including BiomedBERT and GPT-4-1106 Preview, offering an efficient and accurate solution for medical text proofreading [17].

In our study, the best-performing fine-tuned LLMs, Qwen3-14B, outperformed few-shot LLMs and showed performance approaching that of senior radiologists on most metrics under controlled conditions. This demonstrates the strong potential of LLMs in supporting error detection and classification of ultrasound report text across diverse subspecialties. Lightweight open-source LLMs, when fine-tuned with high-quality annotated datasets, can achieve performance comparable to or exceeding that of proprietary LLMs. While enhancing their specialization in clinical information integration and contextual reasoning tasks, open-source LLMs also maintain the deployability and controllability required for clinical implementation. Nevertheless, performance gaps remain for the performance of LLMs on complex reasoning tasks, cross-sentence information integration, and fine-grained error categories, underscoring the importance of continued oversight in clinical deployment to ensure QC accuracy and patient safety. Despite these encouraging results, the observed κ differences indicate that LLM predictions are still less consistent with expert readers than human experts are with each other, suggesting that these systems should currently be considered supportive QC tools rather than replacements for expert review.

From a clinical perspective, several practical considerations should be addressed before deploying LLM-based report quality-control systems in routine practice. Appropriate threshold selection is required to balance sensitivity and false-positive costs, as overly sensitive systems may generate excessive alerts and contribute to alert fatigue. In practice, LLM-based systems hold potential for integration into radiology reporting platforms or PACS as post-report QC tools, offering assistive feedback rather than replacing expert review. Before real-world deployment, prospective validation, ongoing monitoring, and adaptation to institution-specific reporting practices are necessary to ascertain their effects on workflow efficiency, diagnostic safety, and patient outcomes, since the current study did not evaluate downstream clinical impact.

This study has limitations. First, our evaluation focused on error detection and classification within the textual content of ultrasound reports, without systematically assessing cross-modal discrepancies between ultrasound images and textual reports. Future work should investigate whether multi-modal LLMs can detect inconsistencies across the image evidence, text description, and text conclusion. Second, the error patterns and categories predefined in this study were derived from established literature and common mistakes in clinical practice; however, some degree of subjectivity remains inevitable. In real-world practice, ultrasound reports may contain more complex and heterogeneous error patterns, and certain categories may involve borderline cases requiring contextual interpretation. These factors may limit the generalizability of LLMs to previously unseen error patterns. Third, omission errors are inherently more difficult to detect because they involve identifying missing rather than explicit information, which may partly explain the lower performance observed for this category. Finally, all data were derived from Chinese-language ultrasound reports from three institutions within the same region. Variations in reporting conventions, terminology, and narrative styles across institutions, regions, and languages may restrict the generalizability of the present findings. Consequently, further validation in other relatively small ultrasound subspecialties, multi-regional environments, and diverse linguistic contexts is essential to comprehensively evaluate the stability and robustness of LLMs.

In conclusion, fine-tuned open-source LLMs for Chinese ultrasound report QC demonstrate promising potential for clinical translation in terms of accuracy, stability, and deployability. With large-scale clinical validation and continued optimization of human-AI collaborative workflows, LLMs could play a significant role in reducing diagnostic errors, improving report consistency, and alleviating clinician workload.

## Supplementary information

ELECTRONIC SUPPLEMENTARY MATERIAL

## Data Availability

Upon approval by the institutional review board Ethics Committee of all participating hospitals, a dataset of individual participant, with all sensitive information de-identified, can be shared for research purposes upon request to the corresponding author (L.S.).

## Abbreviations

LLM: Large language models
PPV: Positive predictive value
QC: Quality control
TPR: True positive rate
