# Supplementary material for: From zero-shot to fine-tuning: optimize large language models for error detection of ultrasound reports
Source: Insights Imaging. 2026 Sep 5;17:224. doi: 10.1186/s13244-026-02394-2 (PMC13546399; doi:10.1186/s13244-026-02394-2)
Supplement: Supplementary file 1 [file 13244_2026_2394_MOESM1_ESM.pdf]

From Zero-Shot to Fine-Tuning: Optimize Large Language Models for Error Detection of Ultrasound Reports

ELECTRONIC SUPPLEMENTARY MATERIAL

Insights Imaging (2026) Min L, Jin Z, Yaer L et al.

Appendix content

Appendix Methods 1. Inference configuration and prompt templates of proprietary large language models

Appendix Methods 2. Detailed training parameters of open-source large language models

Appendix Figure 1. Constructed example illustrating all six predefined error categories, along with the corresponding detection results of all large language models under different settings

Appendix Figure 2. Performance metrics of proprietary LLMs in the zero shot and few shot settings

Appendix Figure 3. Performance metrics of nine proprietary LLMs (GPT-4o, GPT-4.1, GPT-o3, Claude 3.5, Gemini 2.5 Pro, DeepSeek-V3, DeepSeek-R1, Grok-3, Grok-4) and seven open-source LLMs (Qwen3-14B, Qwen1.5-7B, Baichuan2-13B, Baichuan2-7B, Mistral-7B, DeepSeek-7B, LLaMA2-7B) under few-shot and fine-tuned settings, respectively

Appendix Figure 4. Training and validation loss curves of fine-tuned open-source LLMs

Appendix Figure 5. Cohen's κ agreement heatmap between all LLMs and radiologists

Appendix Figure 6. Forest plot of the performance distribution

Appendix Figure 7. Pairwise p-values for detection accuracy of all large language models and radiologists

Appendix Figure 8. Pairwise p-values for Macro-F1 score of all large language models and radiologists

Appendix Figure 9. Pairwise p-values for positive predictive value of all large language models and radiologists

Appendix Figure 10. Pairwise p-values for true positive rate of all large language models and radiologists

Appendix Figure 11. Distribution of F1-scores for identifying a specific error category by different zero-shot proprietary large language models

Appendix Figure 12. Distribution of F1-scores for identifying a specific error category by different few-shot proprietary large language models

Appendix Figure 13. Distribution of F1-scores for identifying a specific error category by different fine-tuned open-source large language models

Appendix Figure 14. Distribution of F1-scores for identifying a specific error category by

radiologists with different levels of experience

Appendix Figure 15. Venn diagrams in error predictions among three top-performing large language models

Appendix Table 1. Basic information of nine proprietary large language models

Appendix Table 2. Basic information of the seven open-source large language models

Appendix Table 3. Baseline characteristics across train, validation, test, and natural-error datasets

Appendix Table 4. Performance of zero-shot proprietary LLMs, few-shot proprietary LLMs, fine-tuned open-source LLMs, and radiologists on the natural error cohort

Appendix Methods 1. Inference configuration and prompt templates of proprietary large language models

1.1 Inference hyperparameters

As proprietary LLMs cannot be deployed locally, all inference was conducted via their respective official web platforms or API endpoints. The GPT series and Claude 3.5 Sonnet were accessed through the OpenAI and Anthropic APIs, respectively, whereas Gemini 2.5 Pro, the DeepSeek series, and the Grok series were accessed through the official Google, DeepSeek, and xAI consoles or APIs. To ensure stable, well-structured, and comparable outputs from the Chinese ultrasound report quality control task, a unified prompt format was used across all models. Inference hyperparameters were standardized as follows: temperature = 0.2 to minimize randomness and enhance stability, top_p = 0.9 to balance diversity, and max_tokens = 1024 to limit output length. The number of examples (n_shots) depended on the inference paradigm: zero-shot used 0 examples, while few-shot used exactly 7 examples (including 1 correct report and 6 erroneous reports). The stop parameter was set to ["\n\n"], configured according to each API interface's specification.

All inputs and outputs were processed and recorded through standardized Python scripts to ensure uniform formatting, token budget, and output structure across models. All model responses were automatically parsed into structured results for subsequent evaluation of accuracy and error classification.

1.2 Zero-shot prompt template (JSON example)

{

"system": "You are a senior ultrasound specialist with extensive experience in quality control of Chinese ultrasound reports, including knowledge of standardized ultrasound terminology, structural conventions, and common error patterns.",

"user": "Please review the following Chinese ultrasound report and identify any existing errors, specifying the corresponding error types. The six error types include: (a) omission: the omission of relevant structures or expressions that should be reported; (b) redundancy: inclusion of irrelevant structures or unnecessary repetition; (c) unit or numerical: missing or incorrect measurement units, or implausible numeric values; (d) spelling or expression: misspellings, grammatical mistakes, or semantically incoherent phrasing; (e) logic: lack of a coherent reasoning chain between "ultrasound findings" and "ultrasound indications", resulting in unsupported inferences (excluding orientation errors); and (f) orientation: discrepancies in lesion location descriptions between "ultrasound findings" and "ultrasound indications".\n\n[Ultrasound Examination Items]\n...\n\n[Ultrasound Description]\n...\n\n[Ultrasound Impression]\n...",

"expected_output_format": "Return only a single-line JSON object matching this schema:\n{\n \"omission\":0/1, \"redundancy\":0/1, \"unit_value\":0/1,\n \"spelling\":0/1, \"logic\":0/1, \"orientation\":0/1\n}"

}

1.3 Few-shot prompt template (JSON example)

To improve contextual relevance, a customized strategy for selecting example reports rather than fixed exemplars was employed. For each test report, the tailored example was selected using a dual-criteria matching algorithm based on: (1) error-label similarity: preliminary model predictions for the current test report were used to retrieve candidate reports with similar

combinations of error types; and (2) textual similarity: sentence embeddings were computed to identify linguistic style and content similarity, prioritizing semantically similar examples. The detailed inference prompt templates were as follows:

{
"system": "You are a senior ultrasound specialist with extensive experience in quality control of Chinese ultrasound reports, including knowledge of standardized ultrasound terminology, structural conventions, and common error patterns.",
"user": "Please review the following Chinese ultrasound report and identify any existing errors, specifying the corresponding error types. The six error types include: (a) omission: the omission of relevant structures or expressions that should be reported; (b) redundancy: inclusion of irrelevant structures or unnecessary repetition; (c) unit or numerical: missing or incorrect measurement units, or implausible numeric values; (d) spelling or expression: misspellings, grammatical mistakes, or semantically incoherent phrasing; (e) logic: lack of a coherent reasoning chain between “ultrasound findings” and “ultrasound indications”, resulting in unsupported inferences (excluding orientation errors); and (f) orientation: discrepancies in lesion location descriptions between “ultrasound findings” and “ultrasound indications”. The following are reference examples:\n\n[Example 1]\nUltrasound Examination Items: …\nReport: …\nError Type: Logic Error\nError Content: …\n\n[Example 2]\nUltrasound Examination Items: …\nReport: …\nError Type: Measurement/Unit Error\n...\n\n[Example 7]\n...\n\n[Report for Evaluation]\nUltrasound Examination Items: …\nUltrasound Description: …\nUltrasound Impression: …",
"expected_output_format": "Return only a single-line JSON object matching this schema:\n{\n \"omission\":0/1, \"redundancy\":0/1, \"unit_value\":0/1,\n \"spelling\":0/1, \"logic\":0/1, \"orientation\":0/1\n}"
}

1.4 Model adaptation and input strategy

All prompts were formulated using natural language instructions in Chinese. The same zero-shot prompt content was applied uniformly across all models. For few-shot prompting, the example dataset was generated via a rule-based sample-retrieval algorithm, automatically selecting seven representative examples from the validation dataset that were most relevant to the structure or error types of the current test case. These examples were inserted into the user prompt using a line-by-line concatenation format, ensuring consistent structural alignment and avoiding token overflow.

Appendix Methods 2. Detailed training parameters of open-source large language models

To improve training stability and mitigate overfitting in the small-sample setting, several regularization strategies were adopted. The maximum number of training epochs was set to 100, with a batch size of 16. The AdamW optimizer was used with an initial learning rate of 5×10^{-5}, a weight decay of 0.01, and a linear learning rate decay scheduler with warmup to improve convergence stability. The maximum input sequence length was 1024 tokens, and mixed-precision training using 16-bit floating-point representation was employed to improve computational efficiency and resource utilization. LoRA was configured with a rank of 16 and a dropout rate of 0.05 to improve parameter efficiency. Throughout training, loss curves, classification accuracy, and error-type F1 scores were continuously monitored. Early stopping

based on validation dataset performance was applied to prevent overfitting.

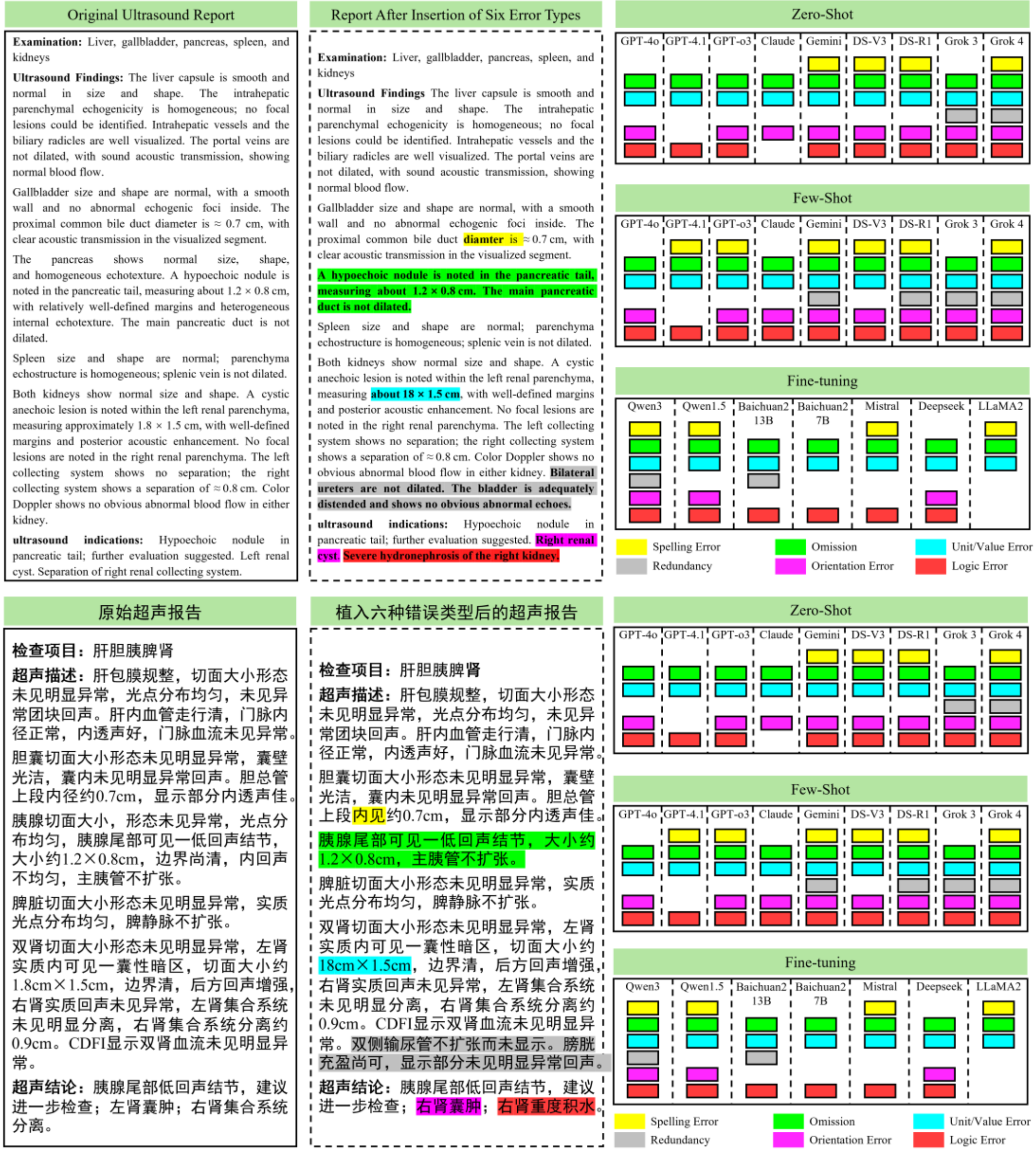

Appendix Figure 1. Constructed example illustrating all six predefined error categories, along with the corresponding detection results of all large language models under different settings. (a) English version; (b) Chinese version. The left panel shows the original quality-controlled report, and the middle panel displays the same report text with the injected errors highlighted using corresponding background colors. The right panel presents the color-coded detection matrix, where each vertical column represents an LLM and each horizontal cell corresponds to one error category. Colored cells indicate successful detection; white cells indicate a miss. As illustrated, fine-tuned LLMs achieve comparable detection performance to few-shot models across all six error dimensions, while relying on smaller model sizes and task-specific adaptation, suggesting improved efficiency and controllability. In contrast, the zero-shot LLMs show more blank cells in the unit/value and orientation error categories, suggesting that purely generalized reasoning remains less effective for numerical verification and laterality determination.

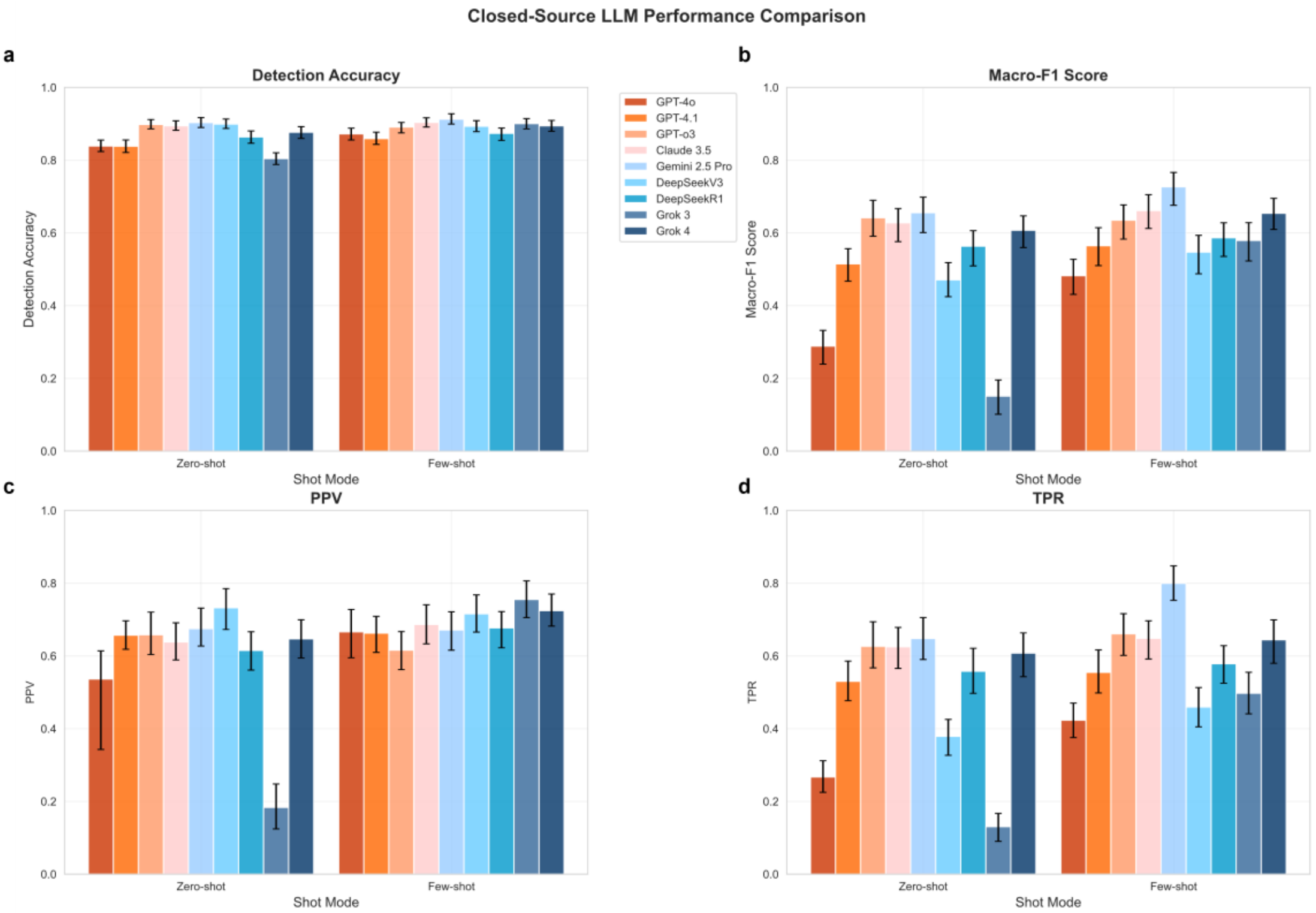

Appendix Figure 2. Performance metrics of proprietary LLMs in the zero shot and few shot settings. (a) Detection Accuracy, indicating whether the model correctly identifies the presence or absence of errors in the ultrasound report; (b) Macro F1 score, reflecting the average classification performance across all error types; (c) Positive predictive value (PPV), representing the proportion of model predicted errors that are true errors; (d) True positive rate (TPR), indicating the proportion of true errors successfully identified by the model. Error bars represent 95% confidence intervals.

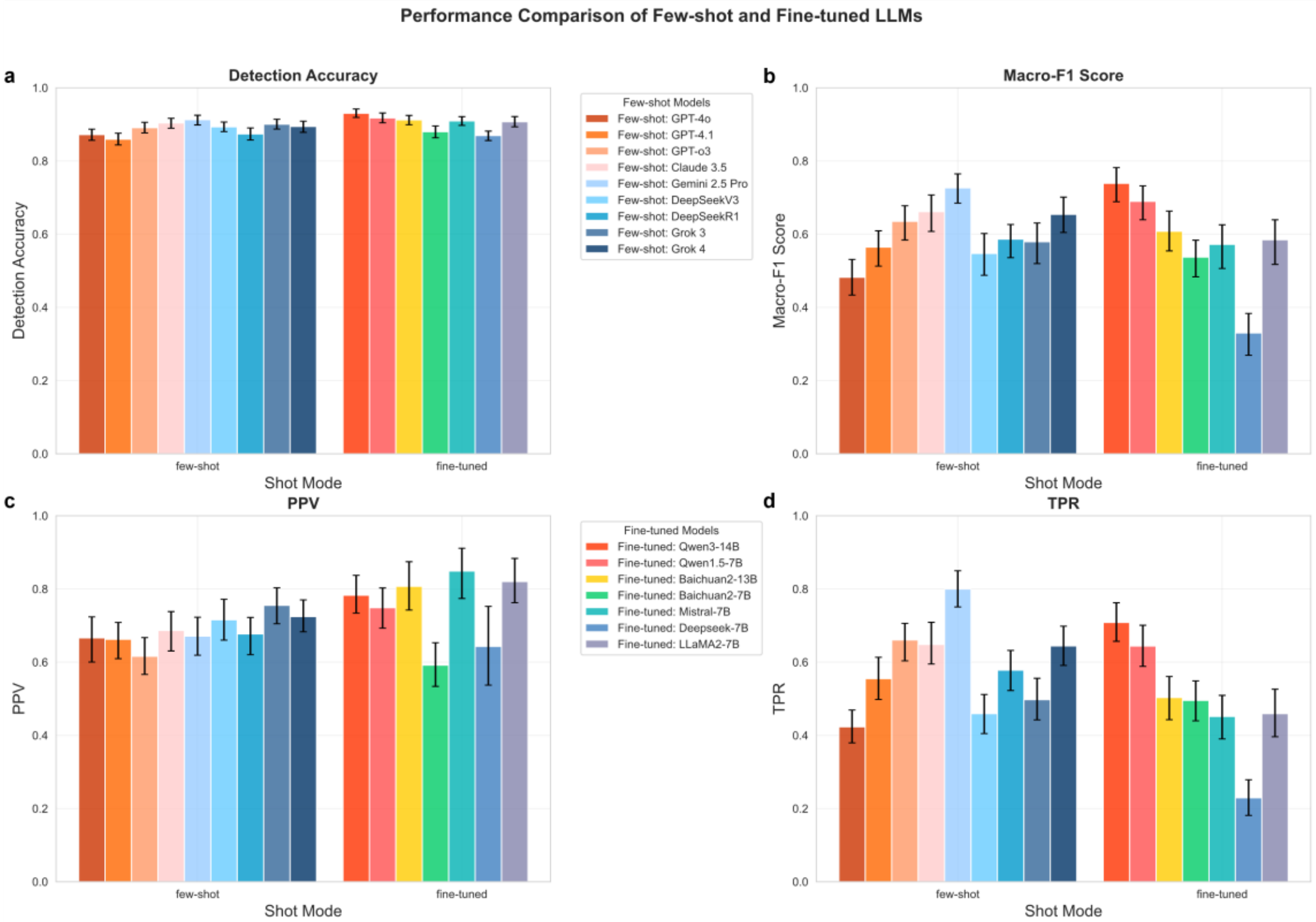

Appendix Figure 3. Performance metrics of nine proprietary LLMs (GPT-4o, GPT-4.1, GPT-o3, Claude 3.5, Gemini 2.5 Pro, DeepSeek-V3, DeepSeek-R1, Grok-3, Grok-4) and seven open-source LLMs (Qwen3-14B, Qwen1.5-7B, Baichuan2-13B, Baichuan2-7B, Mistral-7B, DeepSeek-7B, LLaMA2-7B) under few-shot and fine-tuned settings, respectively. (a) Detection Accuracy, indicating whether the model correctly identifies the presence or absence of errors in the ultrasound report; (b) Macro-F1 score, reflecting the average classification performance across all error types; (c) Positive predictive value (PPV), representing the proportion of model-predicted errors that are true errors; (d) True positive rate (TPR), indicating the proportion of true errors successfully identified by the model. Error bars represent 95% confidence intervals.

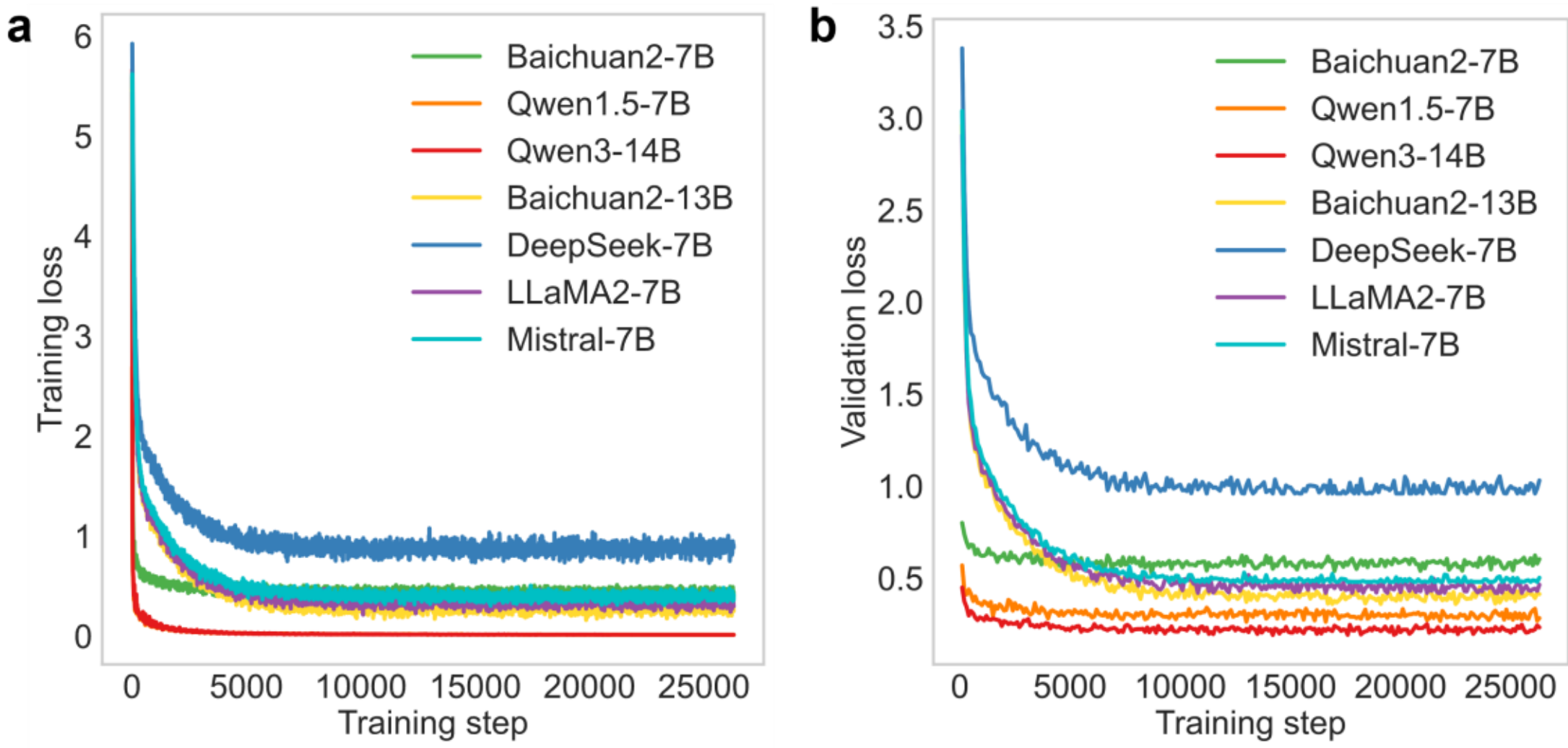

Appendix Figure 4. Training and validation loss curves of fine-tuned open-source LLMs. (a) Training loss across training steps for different models. (b) Validation loss across training steps for different models.

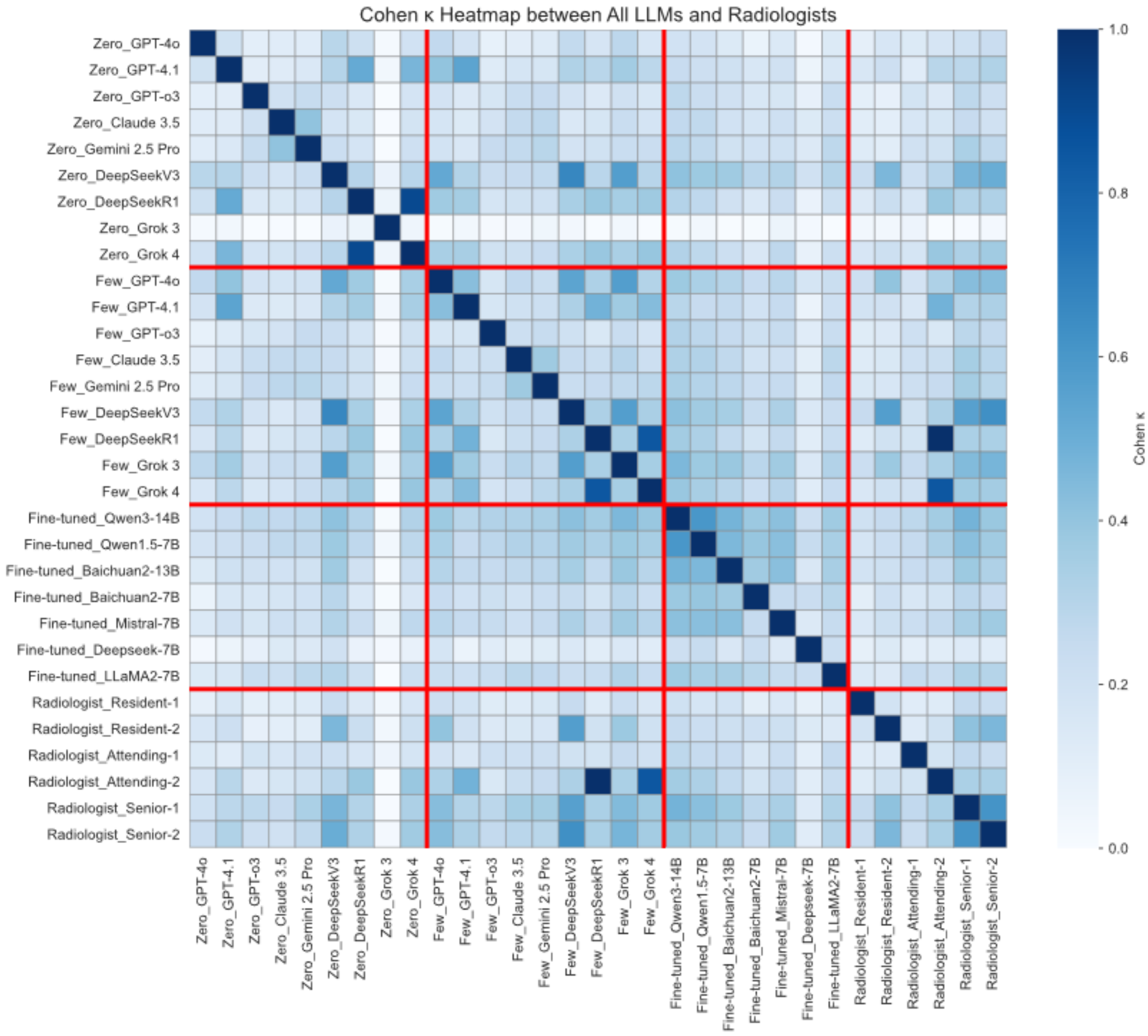

Appendix Figure 5. Cohen's κ agreement heatmap between all LLMs and radiologists. Both the horizontal and vertical axes display 25 groups, comprising LLMs evaluated under 3 paradigms (zero-shot, few-shot, fine-tuned) and 6 radiologists with varying experience levels. Color intensity reflects the magnitude of the Cohen's κ coefficient for prediction in 6 error categories, with darker colors indicating higher agreement and lighter colors indicating lower agreement. Black demarcation lines separate model groups, and red boxes highlight within-group agreement patterns.

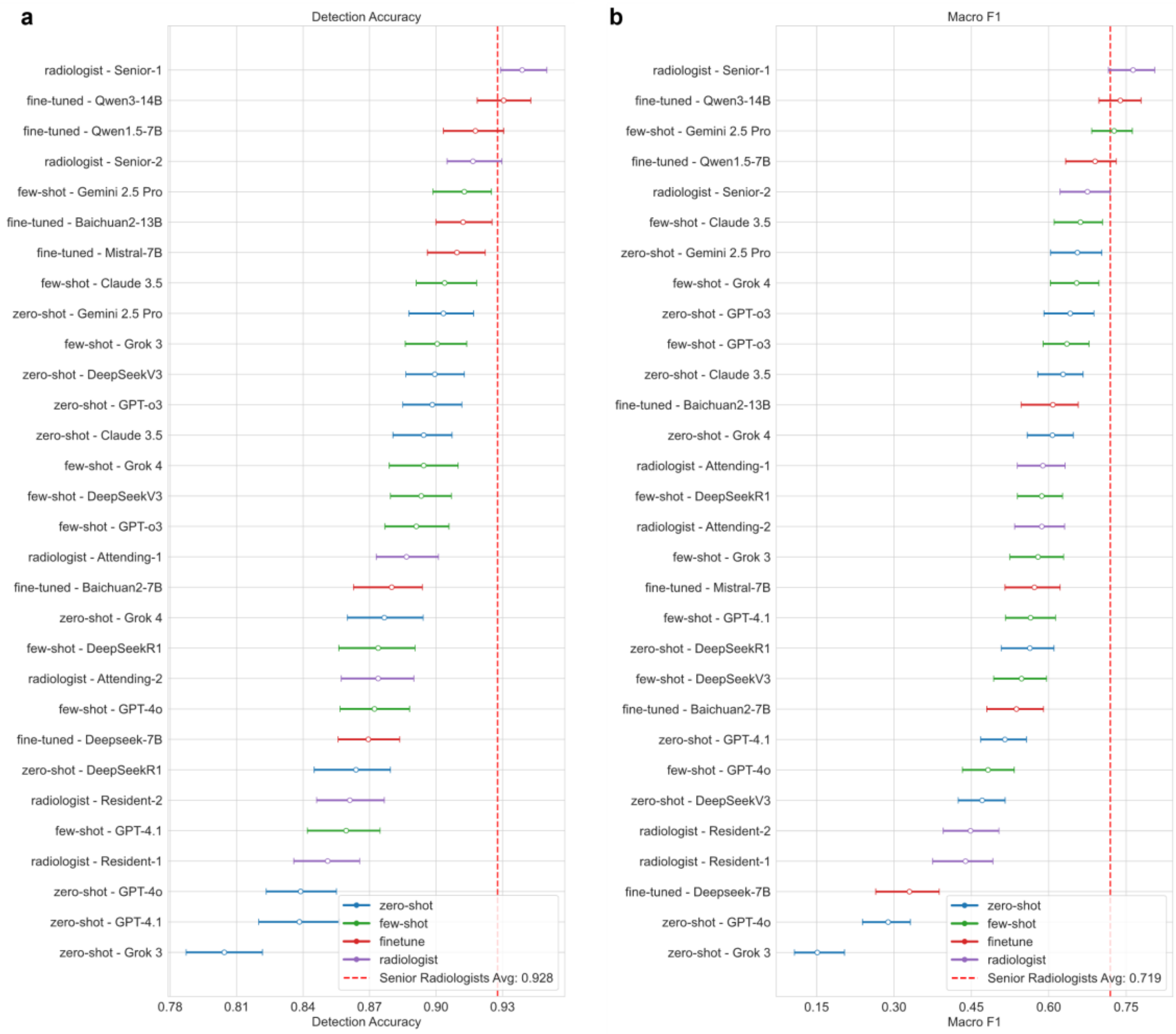

Appendix Figure 6. Forest plot of the performance distribution. (a) Detection accuracy for each large language models in error detection; (b) Macro-F1 for each large language models in error detection. Each dot represents the estimate value, and the horizontal bar denotes the corresponding 95% confidence intervals. Different colors represent different models, including zero-shot, few-shot, fine-tuned, and radiologists. The red dashed line denotes the average performance of senior radiologists, serving as a clinically applicable benchmark in real-world quality control scenarios.

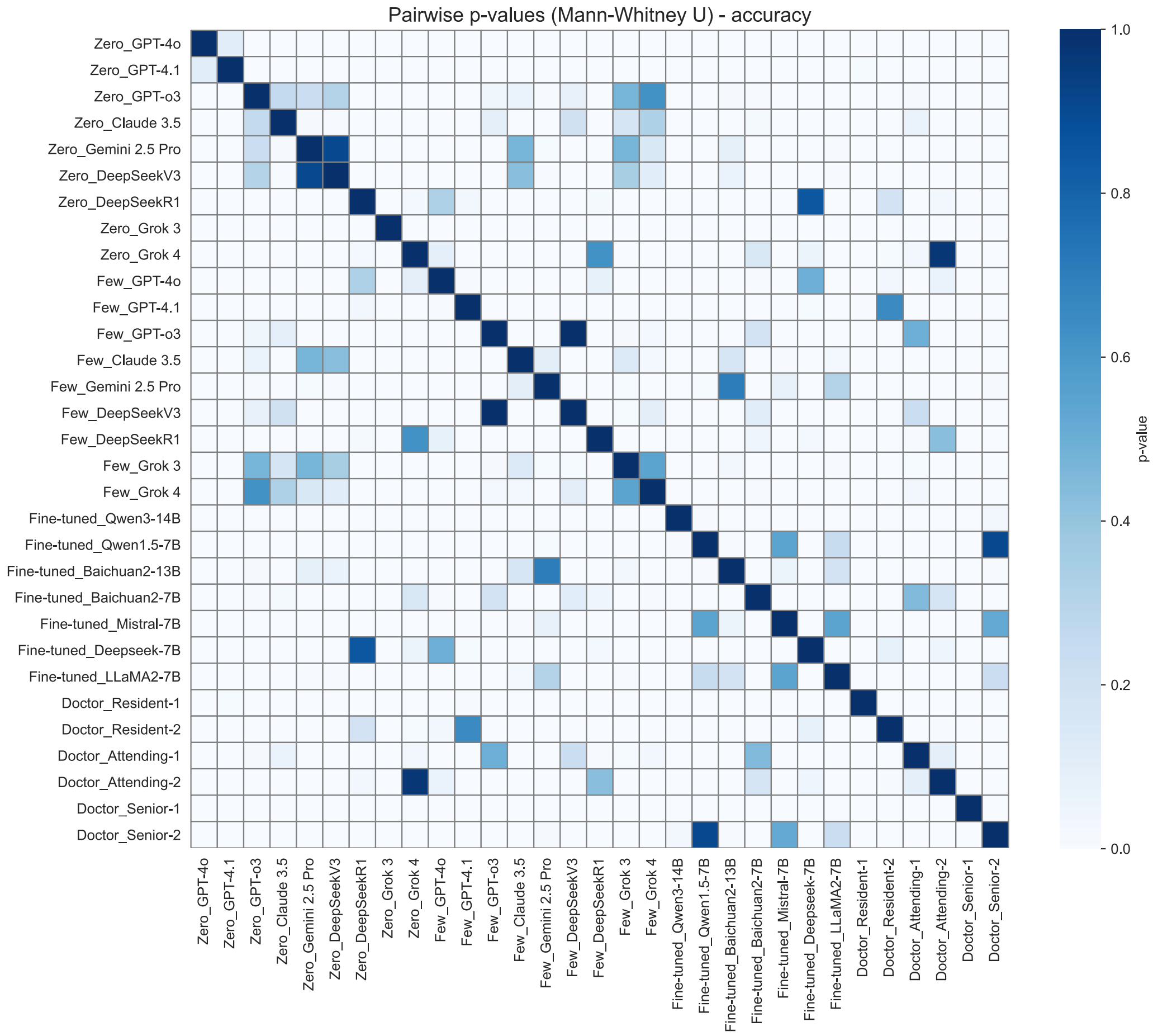

Appendix Figure 7. Pairwise *p*-values for detection accuracy of all large language models and radiologists. This heatmap illustrates pairwise statistical comparisons of detection accuracy across all evaluated LLMs and radiologists. *P*-values were computed using the Mann-Whitney U test based on bootstrapped samples of accuracy scores. Lighter colors indicate lower *p*-values, suggesting stronger evidence of significant differences between model pairs.

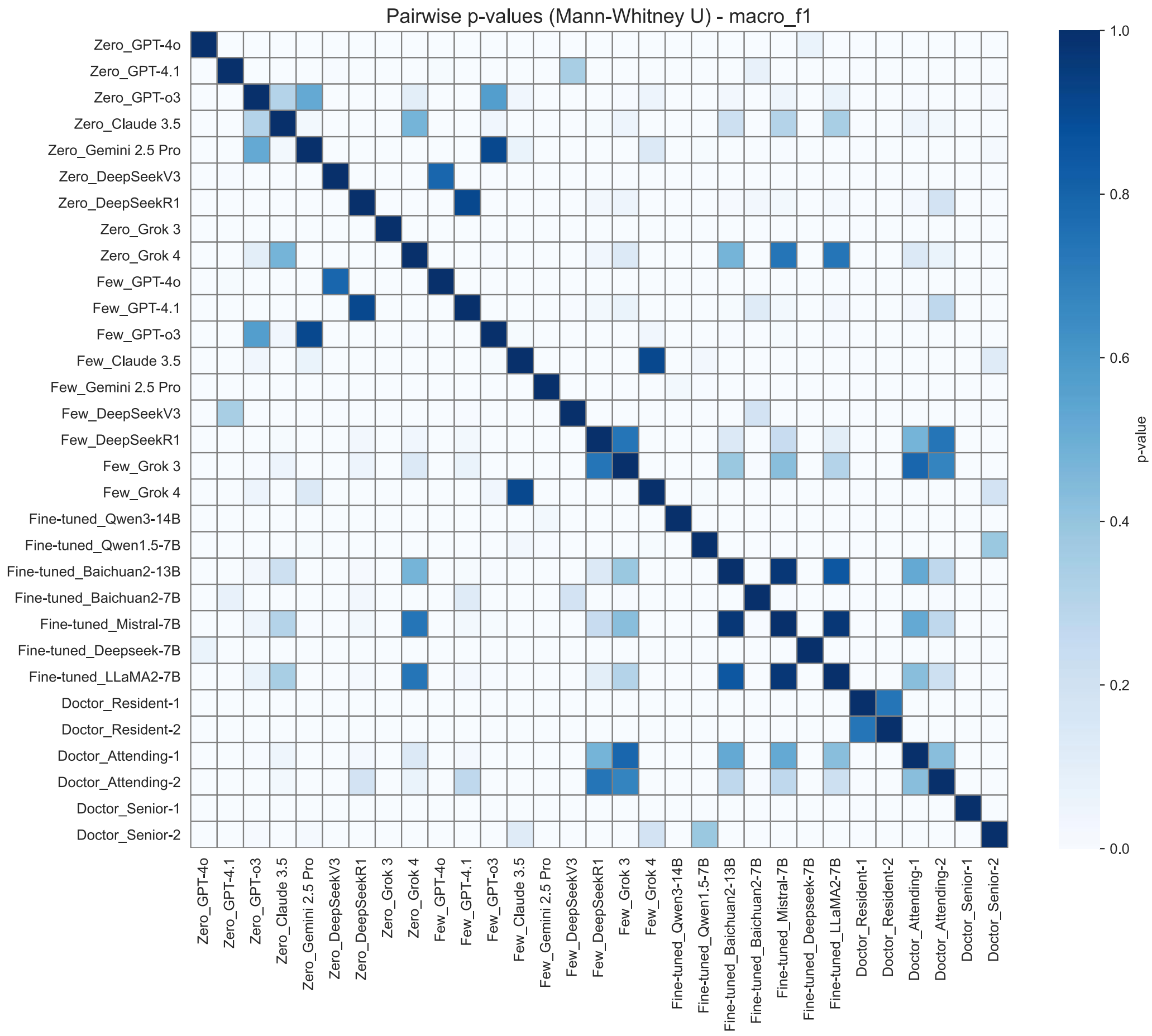

Appendix Figure 8. Pairwise *p*-values for Macro-F1 score of all large language models and radiologists. This heatmap displays the pairwise *p*-values resulting from comparisons of macro-averaged F1-scores between LLMs and radiologists. Each model's performance distribution was generated via bootstrapping. The Mann-Whitney U test was applied to assess the significance of differences. Lighter colors indicate lower *p*-values, suggesting stronger evidence of significant differences between model pairs.

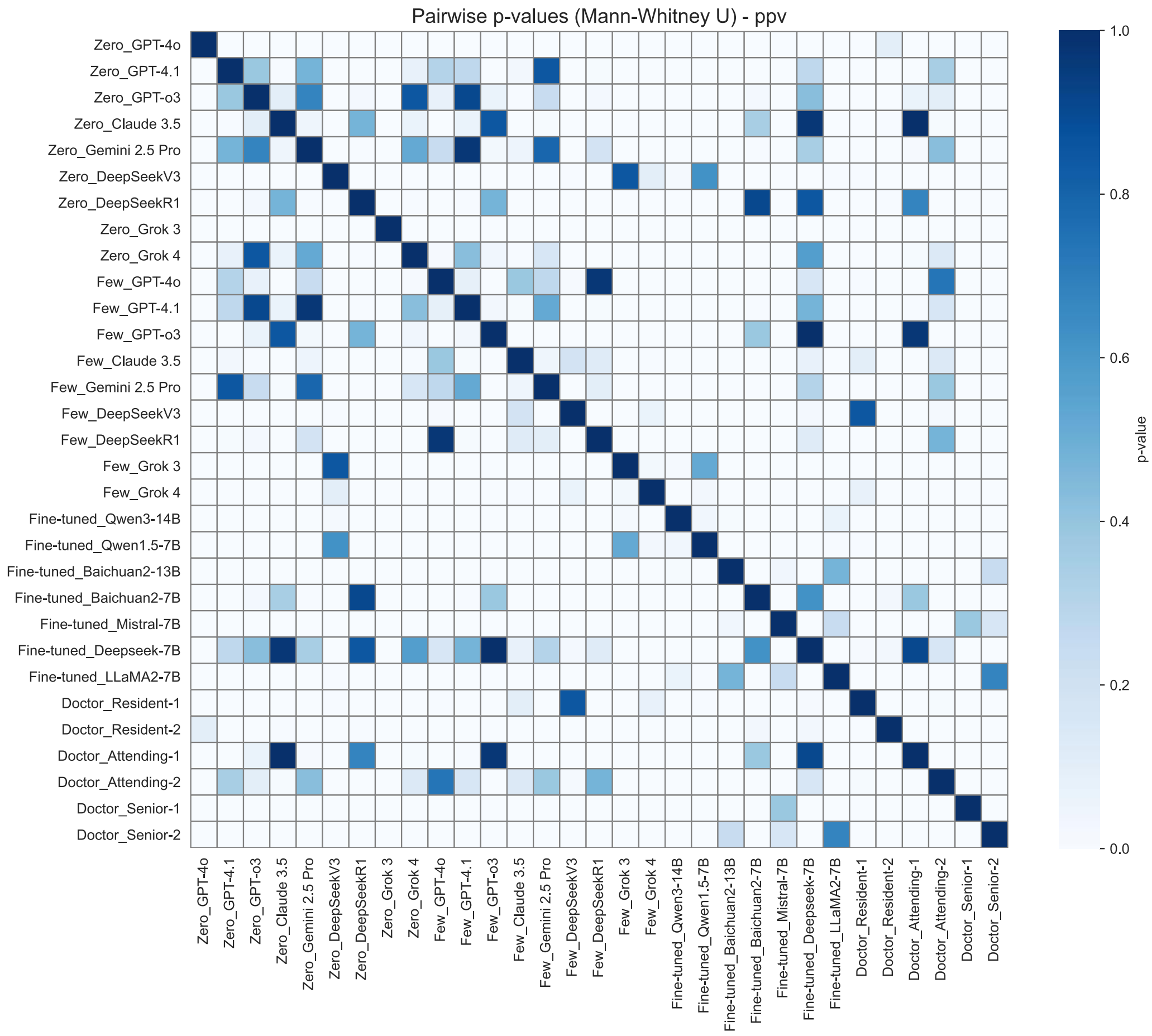

Appendix Figure 9. Pairwise *p*-values for positive predictive value of all large language models and radiologists. The heatmap presents statistical comparisons of positive predictive value (PPV) between all evaluated LLMs and radiologists. Bootstrapped distributions of PPV were used for each model, and the Mann-Whitney U test was applied to calculate the corresponding p-values. Lighter colors indicate lower *p*-values, suggesting stronger evidence of significant differences between model pairs.

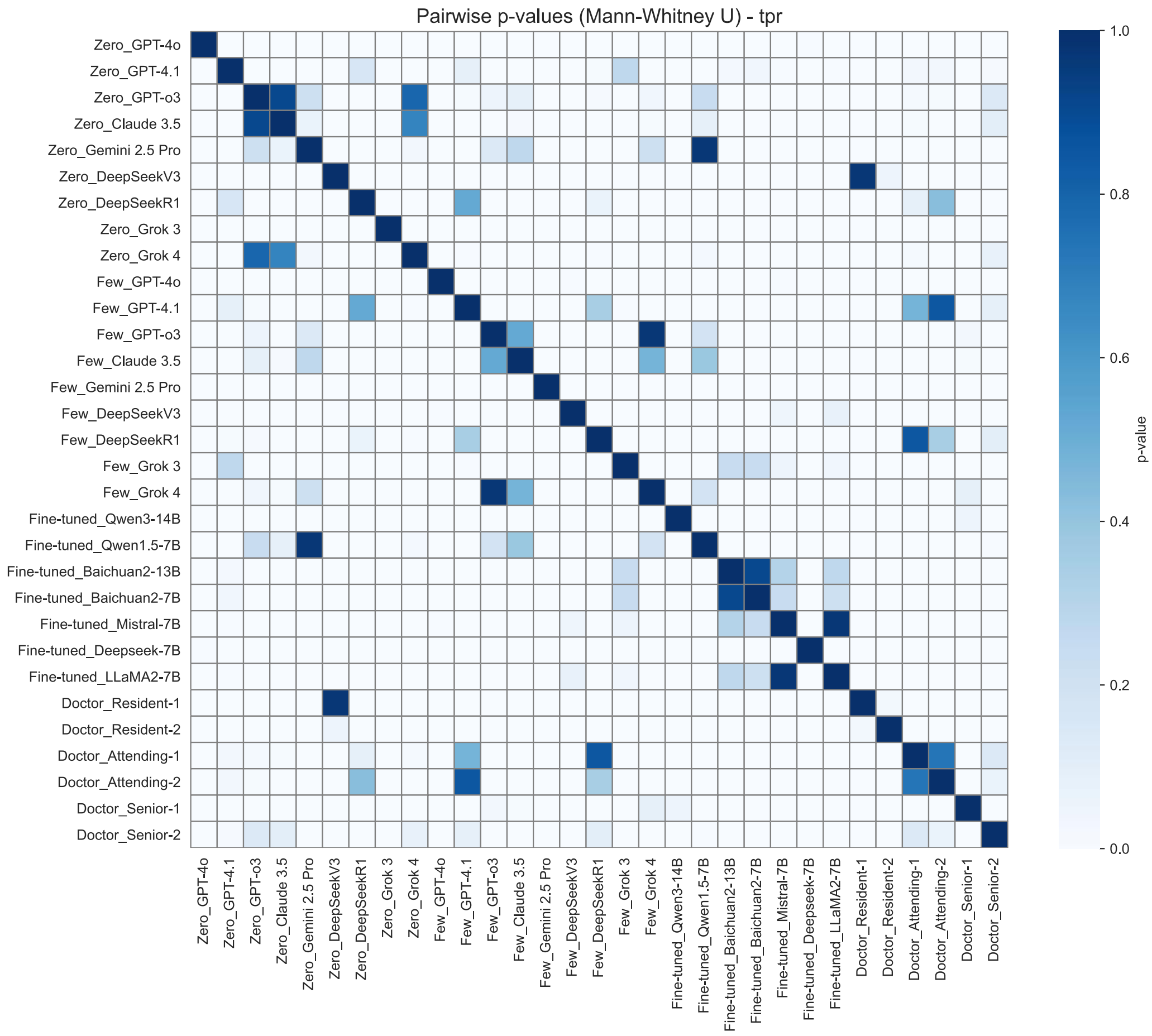

Appendix Figure 10. Pairwise *p*-values for true positive rate of all large language models and radiologists. This figure summarizes the statistical comparisons of true positive rate (TPR) among the evaluated LLMs and radiologists. Each cell represents the p-value from a Mann-Whitney U test between two models, based on bootstrapped estimates of TPR. Lighter colors indicate lower *p*-values, suggesting stronger evidence of significant differences between model pairs.

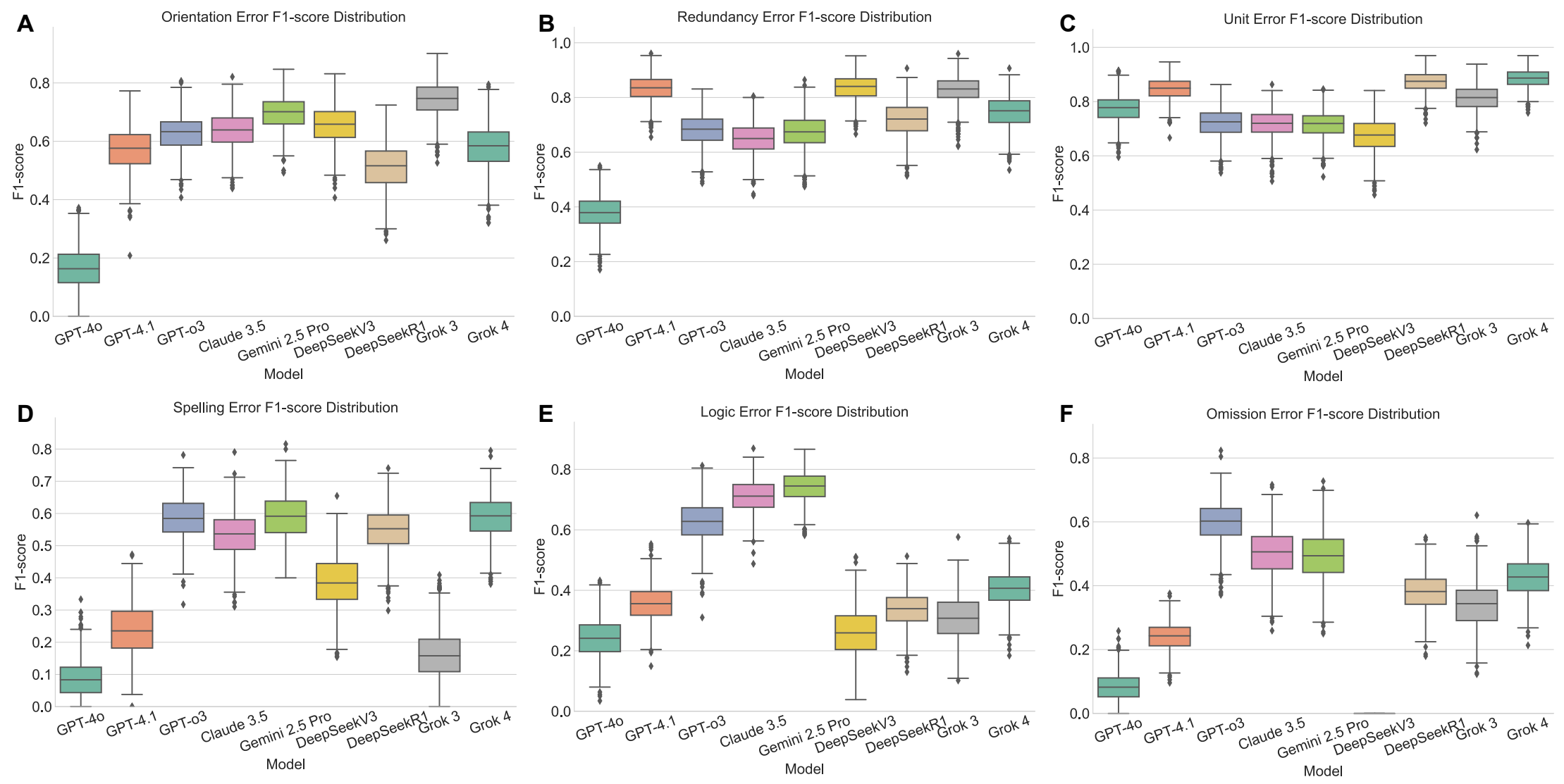

Appendix Figure 11. Distribution of F1-scores for identifying a specific error category by different zero-shot proprietary large language models. (A) omission error, (B) redundancy error, (C) unit/value error, (D) spelling error, (E) logic error, and (F) orientation error. The central box represents the interquartile range (IQR), spanning from the 25th percentile (Q1) to the 75th percentile (Q3) of the F1-scores obtained across all test cases. The horizontal line inside the box indicates the median, reflecting the central tendency of the model performance. A taller box indicates greater variability in F1 scores, whereas a shorter box suggests more consistent performance across cases. The vertical position of the box and whiskers reflects the overall level of performance, with higher positions indicating better detection ability for the given error category.

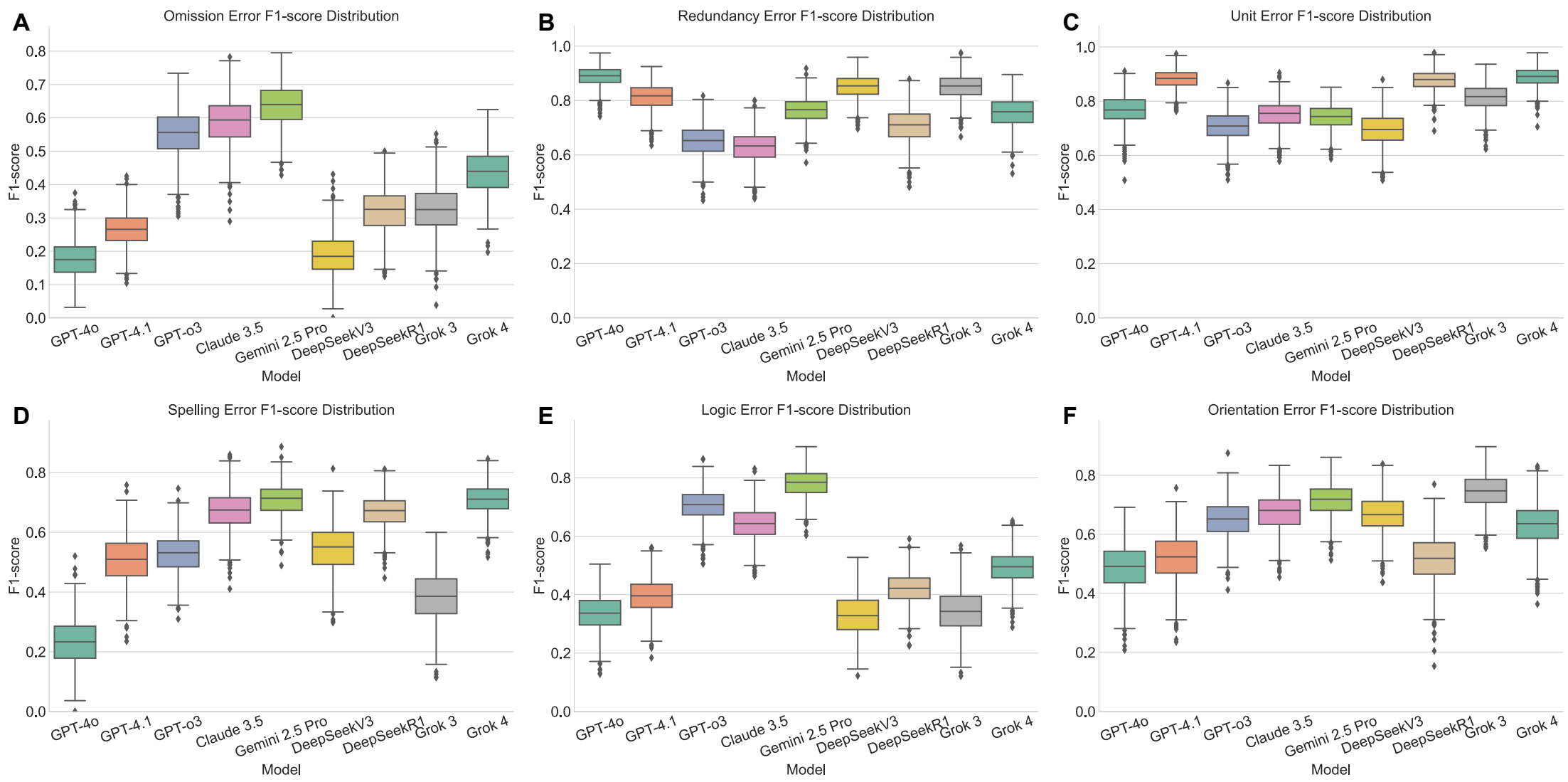

Appendix Figure 12. Distribution of F1-scores for identifying a specific error category by different few-shot proprietary large language models. (A) omission error, (B) redundancy error, (C) unit/value error, (D) spelling error, (E) logic error, and (F) orientation error. The central box represents the interquartile range (IQR), spanning from the 25th percentile (Q1) to the 75th percentile (Q3) of the F1-scores obtained across all test cases. The horizontal line inside the box indicates the median, reflecting the central tendency of the model performance. A taller box indicates greater variability in F1 scores, whereas a shorter box suggests more consistent performance across cases. The vertical position of the box and whiskers reflects the overall level of performance, with higher positions indicating better detection ability for the given error category.

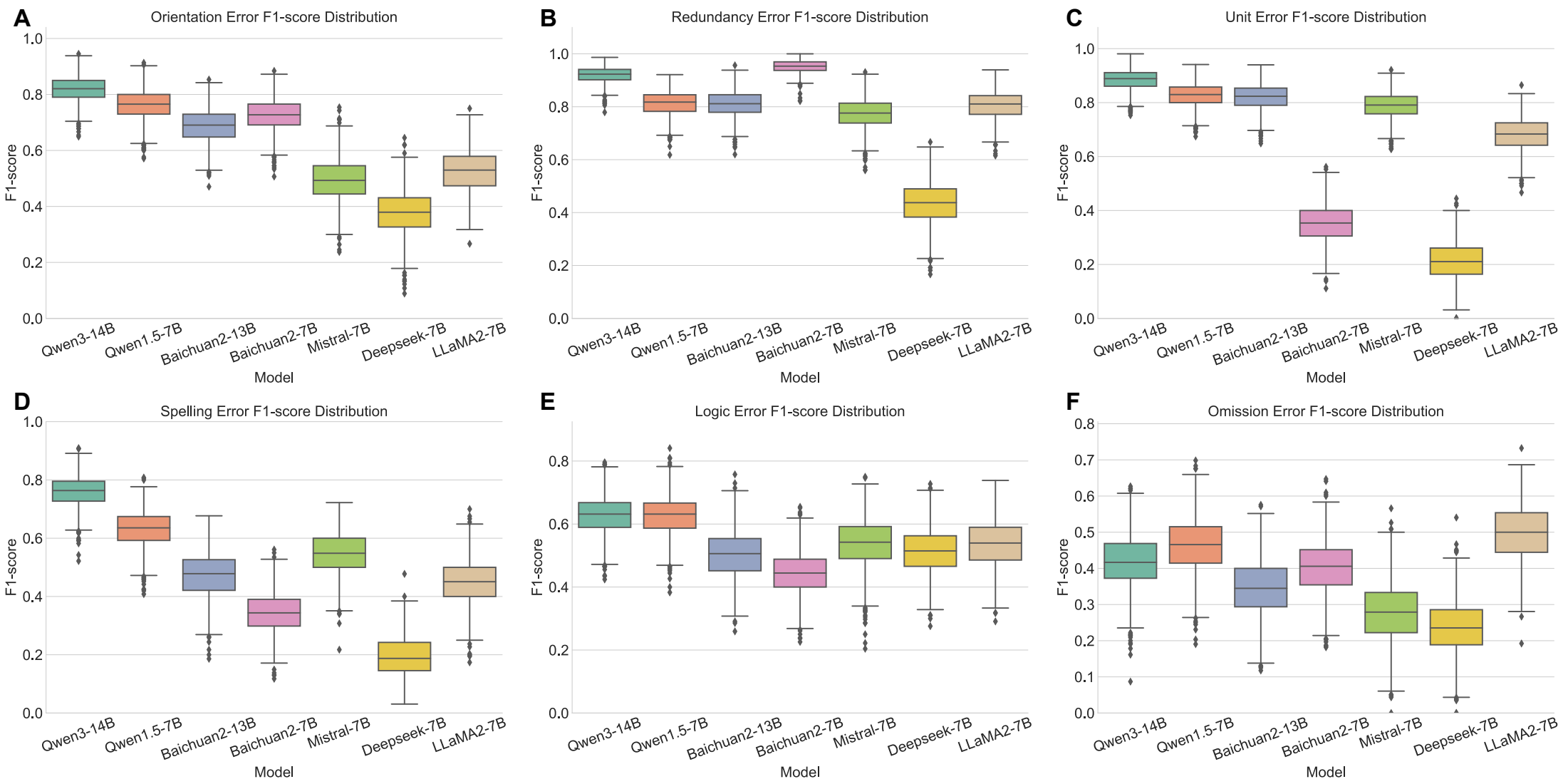

Appendix Figure 13. Distribution of F1-scores for identifying a specific error category by different fine-tuned open-source large language models. (A) omission error, (B) redundancy error, (C) unit/value error, (D) spelling error, (E) logic error, and (F) orientation error. The central box represents the interquartile range (IQR), spanning from the 25th percentile (Q1) to the 75th percentile (Q3) of the F1-scores obtained across all test cases. The horizontal line inside the box indicates the median, reflecting the central tendency of the model performance. A taller box indicates greater variability in F1 scores, whereas a shorter box suggests more consistent performance across cases. The vertical position of the box and whiskers reflects the overall level of performance, with higher positions indicating better detection ability for the given error category.

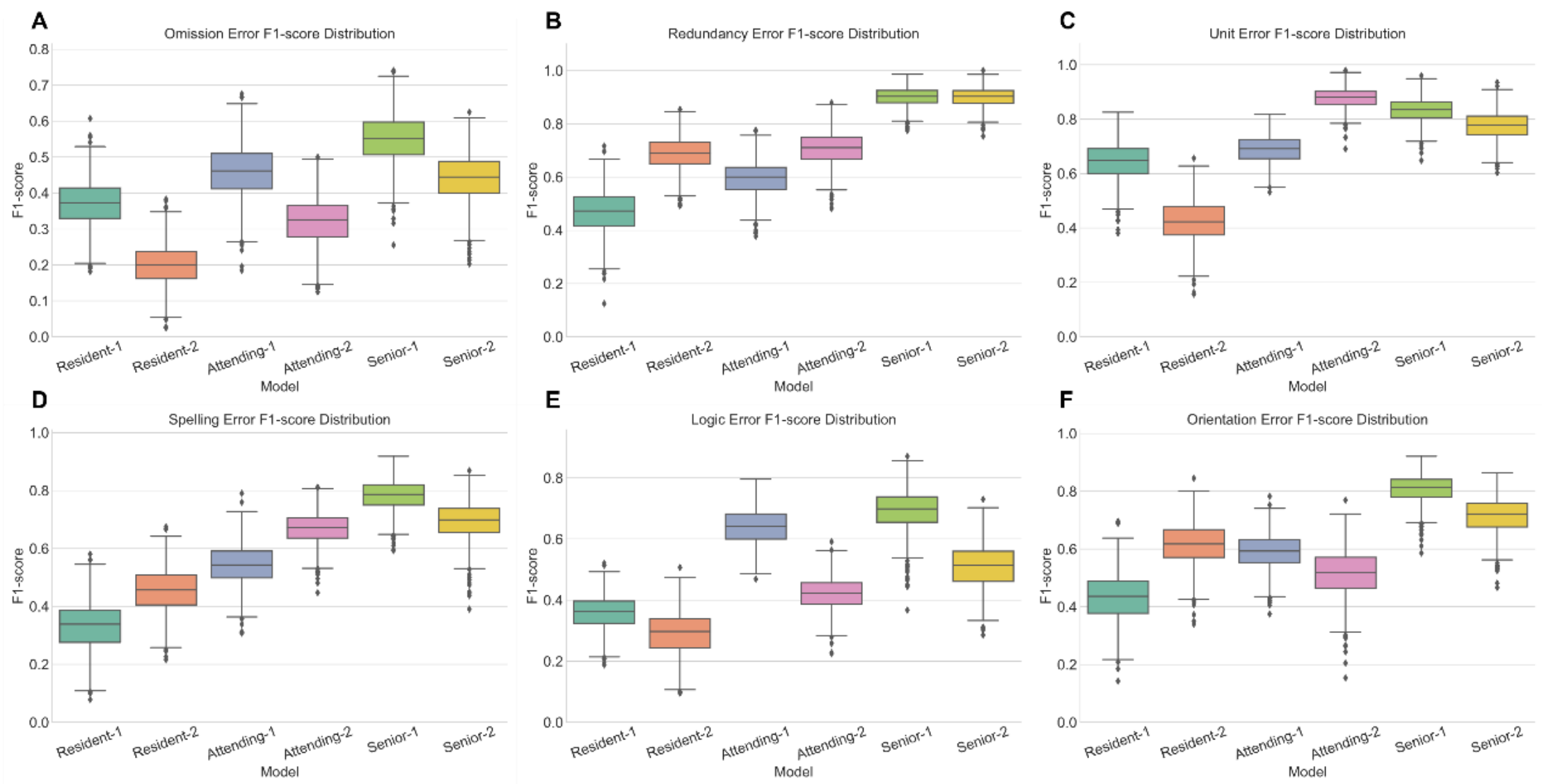

Appendix Figure 14. Distribution of F1-scores for identifying a specific error category by radiologists with different levels of experience. (A) omission error, (B) redundancy error, (C) unit/value error, (D) spelling error, (E) logic error, and (F) orientation error. The central box represents the interquartile range (IQR), spanning from the 25th percentile (Q1) to the 75th percentile (Q3) of the F1-scores obtained across all test cases. The horizontal line inside the box indicates the median, reflecting the central tendency of the model performance. A taller box indicates greater variability in F1 scores, whereas a shorter box suggests more consistent performance across cases. The vertical position of the box and whiskers reflects the overall level of performance, with higher positions indicating better detection ability for the given error category.

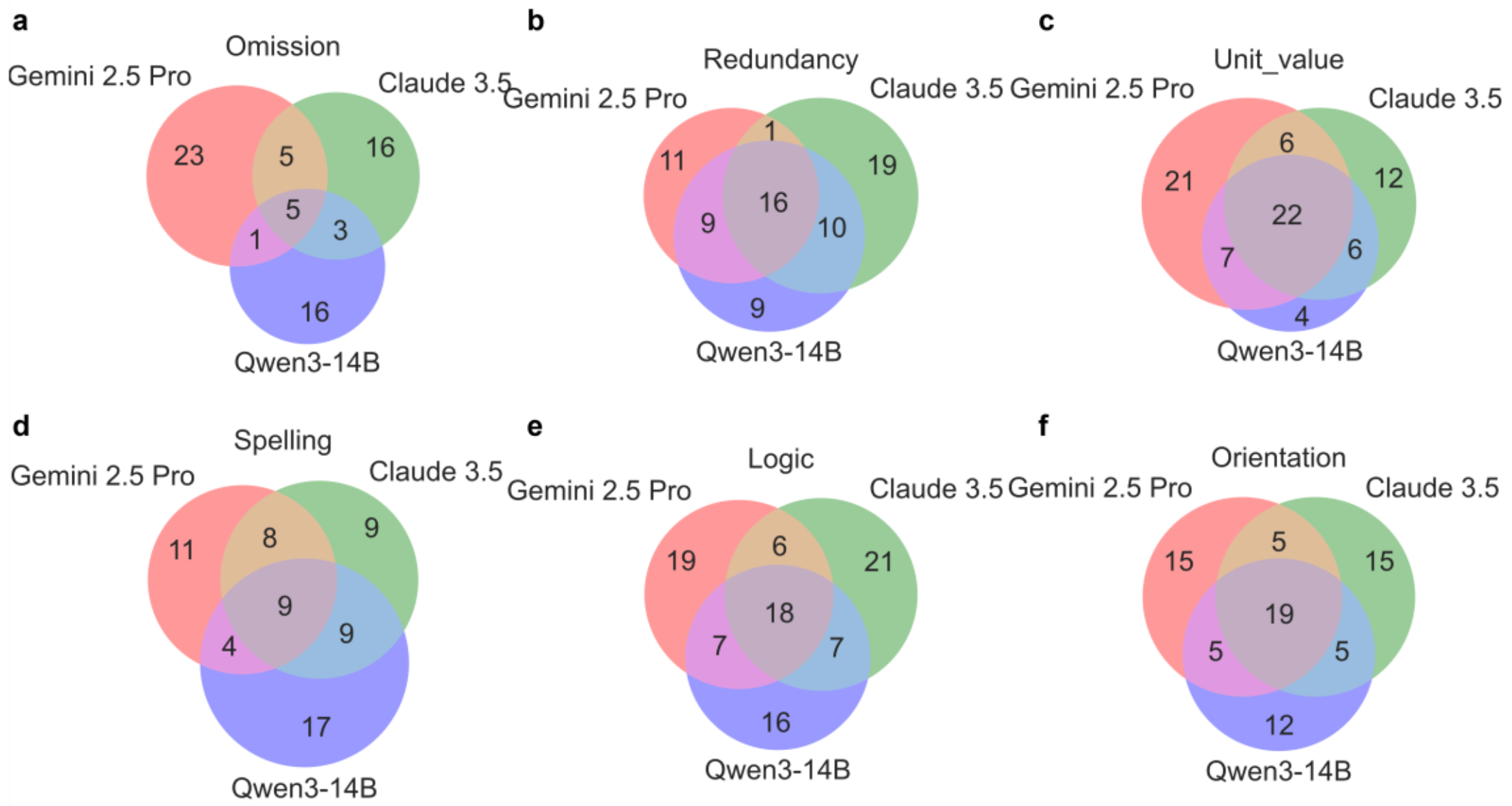

Appendix Figure 15. Venn diagrams in error predictions among three top-performing large language models. a) Omission error, b) Redundancy error, c) Unit/value error, d) Spelling error, e) Logic error, and f) Orientation error. Each colored circle represents the prediction set for a given LLMs, with areas of overlap indicating the degree of concordance in identifying that specific error type

Appendix Table 1. Basic information of nine proprietary large language models

Model Name	Company	Headquarters (Country)	Release Date	Chinese Optimization Support	Website
GPT-4o	OpenAI	United States	May 2024	Yes	https://chat.openai.com/
GPT-4.1	OpenAI	United States	Nov 2023	Yes	https://chat.openai.com/
GPT-o3	OpenAI	United States	Apr 2024	Yes	https://chat.openai.com/
Claude 3.5 Sonnet	Anthropic	United States	Jun 2024	Yes	https://claude.ai/
Gemini 2.5 Pro	Google DeepMind	United States	Jun 2024	Yes	https://gemini.google.com/
DeepSeek-V3	DeepSeek	China	Jul 2024	Yes (native Chinese)	https://chat.deepseek.com/
DeepSeek-R1	DeepSeek	China	Jul 2024	Yes (native Chinese)	https://chat.deepseek.com/
Grok-3	xAI	United States	Feb 2025	Yes	https://x.ai/
Grok-4	xAI	United States	Jul 2025	Yes	https://x.ai/

Appendix Table 2. Basic information of the seven open-source large language models

Model Name	Parameter Scale	Architecture	Release Organization	Release Date	Open-source Platform & URL
Qwen1.5-7B	7B	Transformer	Alibaba Cloud Qwen Team	Dec 2023	github.com/QwenLM
Qwen3-14B	14B	Transformer	Alibaba Cloud Qwen Team	Jun 2024	github.com/QwenLM
Baichuan2-7B	7B	Transformer	Baichuan Intelligence	Sep 2023	huggingface.co/baichuan-inc
Baichuan2-13B	13B	Transformer	Baichuan Intelligence	Sep 2023	huggingface.co/baichuan-inc
Mistral-7B	7B	Dense Transformer + Sliding-Window Attention	Mistral AI	Oct 2023	mistral.ai
DeepSeek-7B	7B	Transformer	DeepSeek-VL Team	Mar 2024	huggingface.co/deepseek-ai
LLaMA2-7B	7B	Transformer	Meta AI	Jul 2023	huggingface.co/meta-llama

Appendix Table 3. Baseline characteristics across train, validation, test, and natural-error datasets

Feature	Category	Train dataset (n=1050)	Validation dataset (n=150)	Test dataset (n=300)	Natural-error dataset (n=300)	*p*-value
Age (years) [a]		52.0 (35.0, 64.0)	54.0 (35.2, 67.0)	53.0 (36.0, 66.0)	54.0 (40.0, 66.0)	0.323
Gender	Female	730 (69.5%)	102 (68.0%)	210 (70.0%)	219 (73.0%)	0.641
	Male	320 (30.5%)	48 (32.0%)	90 (30.0%)	81 (27.0%)	
Medical centers	Center 1	612 (58.3%)	86 (57.3%)	179 (59.7%)	183 (61.0%)	0.100
	Center 2	217 (20.7%)	28 (18.7%)	66 (22.0%)	75 (25.0%)	
	Center 3	221 (21.0%)	36 (24.0%)	55 (18.3%)	42 (14.0%)	
Subgroup	Abdominal	206 (19.6%)	32 (21.3%)	62 (20.7%)	78 (26.0%)	0.006
	Superficial	206 (19.6%)	33 (22.0%)	61 (20.3%)	85 (28.3%)	
	Echocardiography	211 (20.1%)	29 (19.3%)	60 (20.0%)	53 (17.7%)	
	Obstetric and gynecological	213 (20.3%)	29 (19.3%)	58 (19.3%)	53 (17.7%)	
	Interventional	214 (20.4%)	27 (18.0%)	59 (19.7%)	31 (10.3%)	
Error types	Omission	153 (14.6%)	20 (13.3%)	42 (14.0%)	113 (37.7%)	<0.001
	Redundancy	159 (15.1%)	18 (12.0%)	43 (14.3%)	24 (8.0%)	0.014
	Unit_value	161 (15.3%)	17 (11.3%)	47 (15.7%)	31 (10.3%)	0.097
	Spelling	153 (14.6%)	27 (18.0%)	45 (15.0%)	50 (16.7%)	0.631
	Logic	152 (14.5%)	17 (11.3%)	44 (14.7%)	104 (34.7%)	<0.001
	Orientation	142 (13.5%)	19 (12.7%)	42(14.0%)	29 (9.7%)	0.322

[a] Age is reported as the Median (Q1–Q3).

Appendix Table 4. Performance of zero-shot proprietary LLMs, few-shot proprietary LLMs, fine-tuned open-source LLMs, and radiologists on the natural error cohort.

	Detection accuracy	PPV	TPR	Macro F1	MAE$_6$
**Zero-shot LLMs**					
Claude 3.5	0.858 [0.846,0.875]	0.662 [0.626,0.707]	0.553 [0.502,0.632]	0.641 [0.592,0.687]	0.142 [0.125,0.154]
DeepSeekR1	0.830 [0.818,0.843]	0.593 [0.551,0.633]	0.410 [0.354,0.474]	0.532 [0.475,0.590]	0.170 [0.157,0.182]
DeepSeekV3	0.849 [0.840,0.864]	0.812 [0.751,0.874]	0.296 [0.258,0.344]	0.522 [0.471,0.584]	0.151 [0.136,0.160]
GPT-4.1	0.822 [0.808,0.831]	0.566 [0.527,0.603]	0.368 [0.323,0.416]	0.508 [0.456,0.554]	0.178 [0.169,0.192]
GPT-4o	0.814 [0.804,0.824]	0.537 [0.483,0.571]	0.328 [0.287,0.384]	0.469 [0.413,0.519]	0.186 [0.176,0.196]
GPT-o3	0.869 [0.850,0.882]	0.685 [0.640,0.731]	0.613 [0.559,0.658]	0.667 [0.606,0.704]	0.131 [0.118,0.150]
Gemini 2.5 Pro	0.888 [0.869,0.897]	0.716 [0.671,0.754]	0.709 [0.654,0.749]	0.734 [0.685,0.764]	0.112 [0.103,0.131]
Grok 3	0.822 [0.809,0.833]	0.566 [0.521,0.620]	0.368 [0.327,0.418]	0.490 [0.434,0.542]	0.178 [0.167,0.191]
Grok 4	0.856 [0.845,0.873]	0.659 [0.617,0.707]	0.544 [0.495,0.617]	0.635 [0.579,0.697]	0.144 [0.127,0.155]
**Few-shot LLMs**					
Claude 3.5	0.877 [0.865,0.891]	0.679 [0.645,0.714]	0.704 [0.656,0.744]	0.720 [0.673,0.757]	0.123 [0.109,0.135]
DeepSeekR1	0.842 [0.830,0.858]	0.611 [0.572,0.653]	0.524 [0.462,0.572]	0.621 [0.568,0.655]	0.158 [0.142,0.170]
DeepSeekV3	0.857 [0.844,0.871]	0.879 [0.837,0.924]	0.311 [0.251,0.364]	0.554 [0.506,0.620]	0.143 [0.129,0.156]
GPT-4.1	0.828 [0.816,0.841]	0.575 [0.540,0.617]	0.450 [0.401,0.502]	0.579 [0.529,0.626]	0.172 [0.159,0.184]
GPT-4o	0.826 [0.817,0.836]	0.568 [0.533,0.603]	0.439 [0.392,0.498]	0.563 [0.516,0.609]	0.174 [0.164,0.183]
GPT-o3	0.885 [0.872,0.898]	0.690 [0.654,0.728]	0.744 [0.694,0.790]	0.745 [0.700,0.781]	0.115 [0.102,0.128]
Gemini 2.5 Pro	0.899 [0.886,0.913]	0.710 [0.675,0.744]	0.815 [0.767,0.850]	0.784 [0.735,0.817]	0.101 [0.087,0.114]
Grok 3	0.854 [0.843,0.866]	0.637 [0.602,0.679]	0.584 [0.546,0.638]	0.638 [0.585,0.678]	0.146 [0.134,0.157]
Grok 4	0.873	0.672	0.684	0.730	0.127

	[0.863,0.886]	[0.644,0.710]	[0.642,0.744]	[0.688,0.766]	[0.114,0.137]
**Fine-tuned LLMs**					
Baichuan 2-13B	0.898 [0.860,0.929]	0.935 [0.817,1.000]	0.558 [0.381,0.684]	0.728 [0.523,0.828]	0.102 [0.071,0.140]
Baichuan 2-7B	0.866 [0.829,0.913]	0.880 [0.732,1.000]	0.423 [0.279,0.611]	0.631 [0.388,0.783]	0.134 [0.087,0.171]
DeepSeek-7B	0.821 [0.766,0.870]	0.583 [0.425,0.710]	0.538 [0.391,0.684]	0.476 [0.301,0.587]	0.179 [0.130,0.234]
LLaMA2-7B	0.878 [0.844,0.915]	0.923 [0.794,1.000]	0.462 [0.284,0.621]	0.588 [0.259,0.711]	0.122 [0.085,0.156]
Mistral-7B	0.874 [0.846,0.907]	1.000 [1.000,1.000]	0.404 [0.292,0.538]	0.602 [0.401,0.761]	0.126 [0.093,0.154]
Qwen1.5-7B	0.883 [0.872,0.896]	0.885 [0.843,0.915]	0.462 [0.420,0.516]	0.599 [0.559,0.672]	0.117 [0.104,0.128]
Qwen3-14B	0.902 [0.886,0.916]	0.857 [0.794,0.897]	0.595 [0.546,0.648]	0.713 [0.649,0.761]	0.098 [0.084,0.114]
**Radiologists**					
Resident 1	0.818 [0.808,0.830]	0.554 [0.503,0.606]	0.350 [0.310,0.414]	0.482 [0.427,0.540]	0.182 [0.170,0.192]
Resident 2	0.809 [0.798,0.822]	0.519 [0.463,0.563]	0.305 [0.264,0.356]	0.444 [0.386,0.498]	0.191 [0.178,0.202]
Attending 1	0.837 [0.823,0.849]	0.613 [0.564,0.664]	0.447 [0.395,0.503]	0.560 [0.495,0.604]	0.163 [0.151,0.177]
Attending 2	0.844 [0.832,0.855]	0.631 [0.592,0.669]	0.481 [0.430,0.538]	0.587 [0.531,0.628]	0.156 [0.145,0.168]
Senior 1	0.906 [0.891,0.921]	0.739 [0.700,0.784]	0.798 [0.754,0.837]	0.780 [0.737,0.822]	0.094 [0.079,0.109]
Senior 2	0.881 [0.869,0.893]	0.704 [0.670,0.740]	0.672 [0.628,0.717]	0.717 [0.675,0.746]	0.119 [0.107,0.131]

Abbreviations: PPV, positive predictive value; TPR, true positive rate; MAE_6, mean absolute error across six error categories.

Note: Values in brackets represent 95% confidence intervals.
